# SCPP Gene Repertoires in Teleosts and Evolutionary Changes in Bone, Teeth, and Scales

**DOI:** 10.1093/gbe/evag148

**Published:** 2026-06-22

**Authors:** Kazuhiko Kawasaki

**Affiliations:** Department of Anthropology, Pennsylvania State University, University Park, PA 16802, USA

**Keywords:** SCPP genes, evolution of biomineralization, teleost teeth, teleost scales, teleost bone, teleost genome duplication

## Abstract

Secretory calcium-binding phosphoprotein (SCPP) genes are involved in bone, tooth, and scale formation in bony vertebrates, including teleosts. Various studies have investigated teleost SCPP genes to pinpoint genetic changes associated with phenotypic changes in mineralized skeletal elements. However, no comprehensive studies have been described to date. Here, SCPP genes were searched in 63 teleost genome sequences. The chromosomal distribution of individual SCPP genes suggests that all SCPP genes, except one, originally formed four clusters after the teleost genome duplication. Subsequently, various SCPP genes were apparently lost in different lineages, and *scpp1* and *spp1* were the only SCPP genes identified in all investigated teleosts. These 63 teleosts include 10 species with reduced scales, 11 scaleless species, and 2 toothless species. Among them, either *scpp7* or *gsp37* was missing or non-functional in 9 species with reduced scales, and both *scpp7* and *gsp37* in all 11 scaleless species. In seahorse and pipefish, which have bone but lost teeth and scales, *scpp1*, *spp1*, and only two other SCPP genes were found, while in closely related scaleless but toothed cornetfish, *scpp5*, *scpp3*, and *odam* were additionally identified. These findings suggest vital roles of *scpp1* and *spp1* in bone, *scpp7* and *gsp37* in scale, and *scpp5*, *scpp3*, and *odam* in tooth formation. However, many other SCPP genes are also involved in the formation of mineralized tissues and are likely important for some specific features of teeth or scales. It appears that the repertoire of SCPP genes in teleost species underlies diverse conditions of mineralized skeletal elements.

## Graphical Abstract

**Graphical Abstract:**
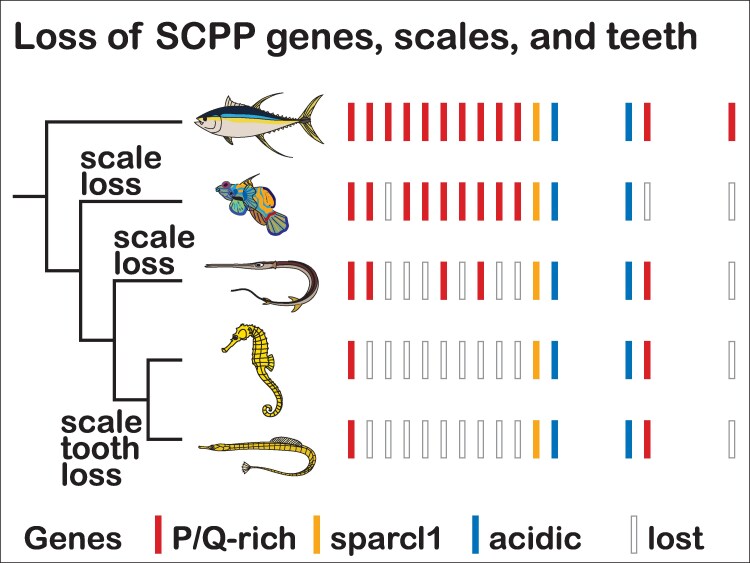

Significance Among many genes participating in tissue mineralization of bony vertebrates, evolutionarily related Secretory Calcium-binding Phospho-Protein (SCPP) genes are especially important because various SCPP genes are involved in the formation of distinct mineralized elements: bone, teeth, and scales. While critical roles of mammalian SCPP genes in the formation of bone and teeth have been extensively investigated, the diversity of SCPP genes is largely unexplored in teleost fishes, the most diversified group of vertebrates. The present investigation into the genome sequence of 63 teleost fishes—including scaleless and toothless species—suggests distinct sets of SCPP genes vital to bone, tooth, or scale formation in teleosts, which differ considerably from SCPP genes involved in the mineralization of bone and teeth in mammals.

## Introduction

Bone, teeth, and scales are principal mineralized skeletal elements that evolved in vertebrates (Hall 2015). Mineralization of skeletal elements involves various extracellular matrix proteins. In bony vertebrates (osteichthyans), many of these proteins belong to the secretory calcium-binding phosphoprotein (SCPP) family (Kawasaki and Weiss 2003). Based on the amino acid composition, SCPPs can be classified into acidic SCPPs and Pro/Gln(P/Q)-rich SCPPs (Kawasaki et al. 2011). In humans, 23 genes encoding SCPPs have been identified. Among them, five genes encode acidic SCPPs that are involved in the formation of bone, dentin, or cementum, while seven genes encode P/Q-rich SCPPs that compose the enamel matrix or enamel–epithelium junction (Chun et al. 2025). Most of these SCPP genes are found in coelacanth and other lobe-finned vertebrates (sarcopterygians) that have enamel on teeth (Kawasaki and Amemiya 2014; Mikami et al. 2022).

SCPP genes are also found in ray-finned vertebrates (actinopterygians). However, the repertoire of SCPP genes differs considerably between sarcopterygians and actinopterygians. Among non-teleost actinopterygians, 39 to 43 SCPP genes were identified in bichirs and gars that possess ganoid scales (covered with ganoin that is equivalent to dental enamel), while 22 to 24 SCPP genes were found in sturgeon, paddlefish, and bowfin, which lost ganoid scales (Mikami et al. 2022). This finding allowed the authors to hypothesize that many SCPP genes were secondarily lost in association with the loss of ganoid scales in sturgeon, paddlefish, and bowfin. Among Teleostei species (teleosts), 23 SCPP genes were identified in zebrafish (Table 1). By contrast, only two or three functional SCPP genes were described in seahorse and pipefish (syngnathids), which led to the hypothesis that the loss of SCPP genes explains the loss of teeth in syngnathids (Lin et al. 2016; Zhang et al. 2020b).

**Table 1:** The number of apparently functional SCPP genes in teleosts and the presence, absence, or reduction of scales and teeth

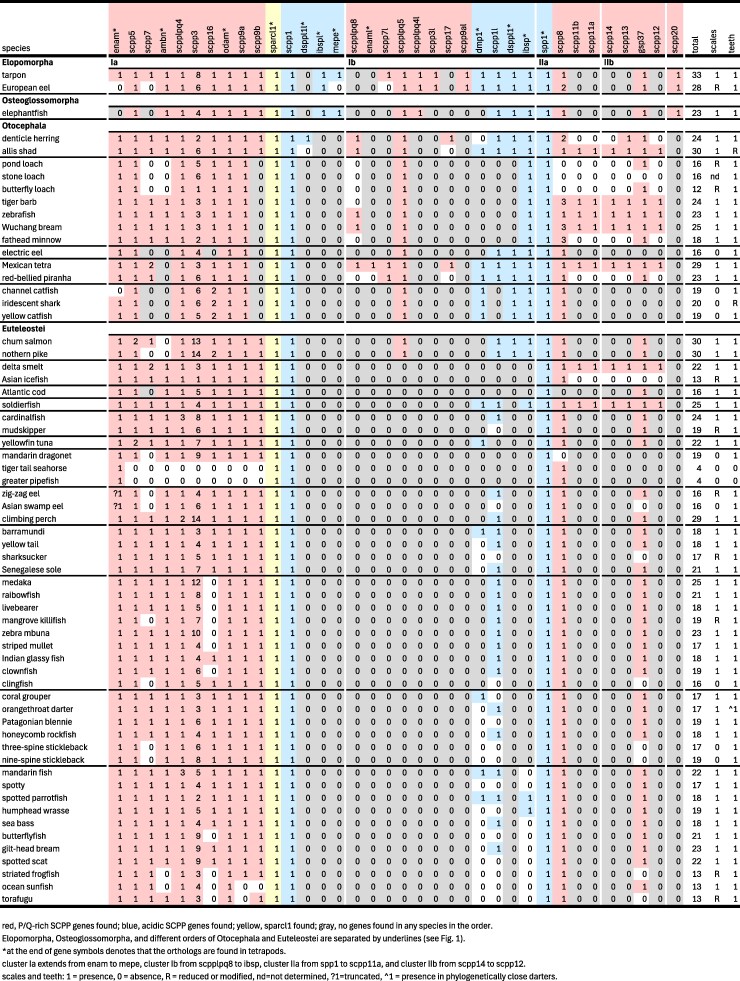

**Fig. 1.**
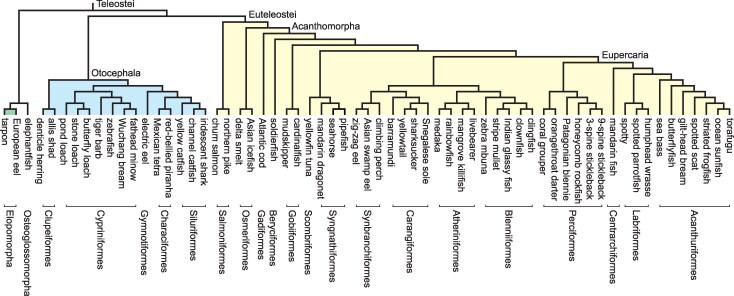
Phylogeny of 63 Teleostei species (teleosts) analyzed in this study. These 63 species encompass Elopomorpha (2 species; green), Osteoglossomorpha (1 species), Otocephala (5 orders, 15 species; blue), and Euteleostei (15 orders, 45 species; yellow). See Table S1 for their Latin names. The phylogeny of these teleosts is based on the phylogenetic classification of ray-finned fishes (Near and Thacker 2024).

Various other studies have also described correlations between changes of mineralized skeletal elements and SCPP gene repertoires in teleosts. To further these hypotheses, I searched for SCPP genes in 63 teleosts (Fig. 1), encompassing Elopomorpha (2 elopomorphs), Osteoglossomorpha (1 osteoglossomorph), Otocephala (15 otocephalans), and Euteleostei (45 euteleosts). Results suggest vital SCPP genes for bone, tooth, and scale formation in teleosts.

## Results

### Chromosomal Distribution of SCPP Genes and Teleost Genome Duplication

**Fig. 2.**
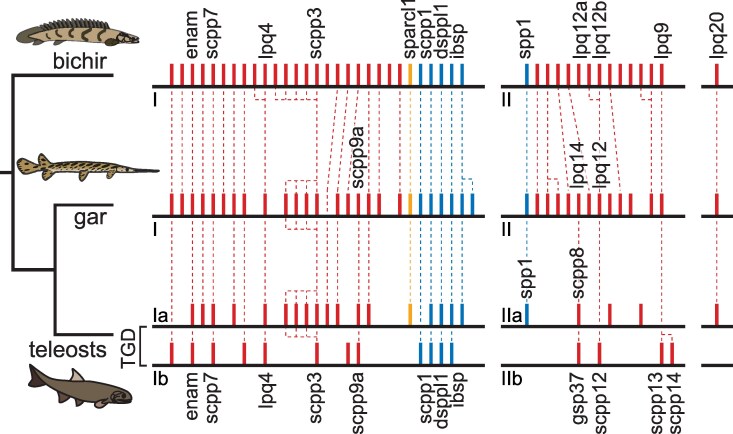
SCPP gene clusters in bichir (*Polypterus senegalus*), gar (*Lepisosteus oculatus*), and a putative ancestral teleost. Each red, yellow, and blue bar represents a P/Q-rich SCPP gene, *sparcl1*, and an acidic SCPP gene, respectively. Orthologs and co-orthologs are connected by dashed lines (multiple *scpp3* genes are also connected by dashed lines). In bichir and gar, all SCPP genes form two clusters I and II, except *scpplpq20* (lpq20) that is located on a different chromosome. The SCPP gene clusters in a putative ancestral teleost were reconstructed by assembling orthologous SCPP genes found in syntenic chromosomal regions of various teleosts, and by removing recently duplicated SCPP genes. For eight SCPP genes, *enam*, *scpp7*, *scpplpq4* (lpq4), *scpp3*, *scpp9a*, *scpp1*, *dsppl1*, and *ibsp*, present in bichir and/or gar cluster I, co-orthologs were found in both teleost clusters Ia and Ib. In addition, for nine SCPP genes present in cluster I, the ortholog was found in either cluster Ia (*scpp5*, *ambn*, *scpp16*, *odam*, and *scpp9b*) or cluster Ib (*scpplpq8*, *scpplpq5*, *dmp1*, and *ibsp*; Table 1). For *scpp17* present in cluster Ib, no clear ortholog was identified in cluster I. Similarly, no clear ortholog of *scpp11b* or *scpp11a* in cluster IIa was identified in cluster II. A previous study suggested that *gsp37* and *scpp8* are co-ortholog of *scpplpq14* (lpq14) (Braasch et al. 2016). The present study proposes that *scpp12* is the ortholog of *scpplpq12* (lpq12; two *scpplpq12* genes are found in bichir), while *scpp13* and *scpp14* are co-ortholog of *scpplpq9* (lpq9; Fig. S2). The result of the present study supports the hypothesis that teleost SCPP gene clusters Ia and Ib, and clusters IIa and IIb arose via the teleost genome duplication (TGD) (Braasch and Postlethwait 2012). Genes forming clusters I and II in bichirs and gars are shown in our previous study (Mikami et al. 2022). See Table 1 for SCPP genes constituting clusters Ia, Ib, IIa, and IIb in the putative ancestral teleost.

Most SCPP genes arose through tandem duplication and initially formed a large gene cluster, which subsequently split into two clusters in the common ancestor of actinopterygians (see clusters I and II in Fig. 2) (Mikami et al. 2022). In teleosts, these SCPP genes underwent the teleost genome duplication (TGD) (Braasch and Postlethwait 2012). To assess the impact of the TGD, the arrangement of SCPP genes was investigated in 63 teleosts. The result showed that, with a few exceptions, all SCPP genes were identified within six or fewer chromosomal regions (Fig. S1, Table S1; see Supplementary Notes for individual SCPP genes). The arrangement of orthologous SCPP genes in each of these chromosomal regions implies that, except for *scpplpq20*, all teleost SCPP genes originally formed four clusters, which are referred to here as Ia, Ib, IIa, and IIb (Fig. 2; see Table 1 for SCPP genes composing each cluster). Although all four clusters, consisting of one or more genes, were found in most teleosts, no SCPP genes originally located in clusters Ib or IIb were identified in some teleosts (Table 1, Fig. S1) presumably because all genes were lost. Cluster Ia contains *sparcl1* (Fig. 2), which is evolutionarily related to SCPP genes (Kawasaki et al. 2017). For *sparcl1* and all SCPP genes in clusters Ia and Ib, except *scpp17*, their orthologs were found in the same order in bichir and/or gar cluster I (Fig. 2). Moreover, eight genes (*enam*, *scpp7*, *scpplpq4*, *scpp3*, *scpp9a*, *scpp1*, *dsppl1*, and *ibsp*) were found in cluster I, cluster Ia, and cluster Ib (Fig. 2). These findings indicate that teleost clusters Ia and Ib are duplicates of the single ancestral cluster that originally formed around *sparcl1*.

Our previous study suggested that SCPP gene clusters IIa and IIb arose via the TGD (Braasch et al. 2016). Cluster IIa resembles cluster II in gar and bichir since all these three clusters include *spp1* (Fig. 2). We previously proposed that *gsp37* and *scpp8* in teleosts are co-orthologs of *scpplpq14* in non-teleost actinopterygians (Kawasaki et al. 2017). Furthermore, the present study detected similar exon–intron structures and weak similarities in encoded amino acid sequences among teleost *scpp13* and *scpp14* genes and gar and bichir *scpplpq9* genes and between teleost *scpp12* genes and gar and bichir *scpplpq12* genes (Fig. 2, Fig. S2). These findings support the hypothesis that teleost SCPP gene clusters IIa and IIb arose through the TGD from an SCPP gene cluster that originally contained *spp1*. Given this scenario, it is also likely that clusters Ia and Ib arose via the TGD. Subsequently, original clusters were diminished or disappeared by the loss of SCPP genes in all teleosts and fragmented by chromosomal rearrangements in some teleosts (Fig. S1). Presumably, *scpplpq20* was also duplicated via the TGD, but one copy remains in elopomorphs and an osteoglossomorph (Table 1).

### Acidic SCPP Genes in Teleosts: Retention and Lineage-specific Loss

Among six acidic SCPP genes (*dmp1*, *scpp1*, *dsppl1*, *ibsp*, *mepe*, and *spp1*) found in bichir and gar, all their orthologs were identified in tarpon. In other teleosts, one or more acidic SCPP genes were missing or nonfunctional except for *scpp1* and *spp1* (Table 1). In all investigated teleosts, *scpp1* was found in cluster Ia, and *spp1* in cluster IIa. In some teleosts, another *scpp1*, *scpp1-like* (*scpp1l*), was additionally identified in cluster Ib.

Two *ibsp* genes were identified in elopomorphs and an osteoglossomorph in clusters Ia (*ibsp-like*; *ibspl*) and Ib (*ibsp*). By contrast, only *ibsp* was identified in otocephalans and euteleosts (Table 1). While *ibsp* was found in all otocephalans investigated, *ibsp* was identified only in Salmoniformes, soldierfish, and wrasses (Tea et al. 2024) among euteleosts. Two *dsppl1* genes, *dsppl1-like* (*dsppl1l*) in cluster Ia and *dsppl1* in cluster Ib, were identified in denticle herring (Table 1). In other teleosts, only *dsppl1* was identified. Within euteleosts, *dsppl1* was found only in Salmoniformes. Among teleosts, apparently functional *dmp1* was found in cluster Ib in elopomorphs, some otocephalans, and six euteleosts (Table 1). The phylogenetic distribution of *dmp1* suggests independent losses of *dmp1* in various teleost lineages. Within teleosts, *mepe* was identified in cluster Ia only in tarpon and elephant fish.

### P/Q-Rich SCPP Genes in Teleosts: Phylogenetic Distribution and Expression in Teeth and Scales

Among P/Q-rich SCPP genes in teleosts, the expression of *enam*, *ambn*, *odam*, *scpp5*, *scpp3*, and *scpp9a* has been detected during tooth formation (Kawasaki et al. 2004; Kawasaki 2009; Morita et al. 2023). Among these genes, *enam* was unidentified, apparently nonfunctional, or truncated in five teleosts, all of which have teeth (Table 1). By contrast, *scpp5*, *scpp3*, and *odam* were identified in all toothed teleosts but not in toothless syngnathids (Lin et al. 2016; Zhang et al. 2020b). Unlike other SCPP genes reported to date, the number of *scpp3* genes varies considerably, and three or more *scpp3* genes were found in most actinopterygians (Table 1, Supplementary Notes). In the NCBI database, six *scpp3* genes of three-d stickleback show extremely high coverages of juvenile tooth plate RNA-seq reads (Table S1), suggesting the production of a large amount of the Scpp3 protein during tooth plate formation. No functional *ambn* was found in various otocephalans and some euteleosts, Salmoniformes, syngnathids, frogfish, or ocean sunfish. By contrast, *scpp9a* was found in all otocephalans investigated and most euteleosts except syngnathids, torafugu, and ocean sunfish.

An *scpp9a*-like gene (*scpp9b*) was found in elopomorphs, an osteoglossomorph, and most euteleosts, but only in Clupeiformes among otocephalans (Table 1). In the NCBI database, *scpp9b* is highly covered with tooth-bearing pharyngeal bone RNA-seq reads in medaka (Table S1). Remarkably, *scpp9b* lost all introns and became a single exon gene in Acanthomorpha (Fig. 1). In the NCBI database, *scpp16* shows a relatively high coverage of the juvenile tooth plate RNA-seq reads in three- stickleback (Table S1). While *scpp16* was found in elopomorphs, an osteoglossomorph, and otocephalans except for electric eel, functional *scpp16* was unidentified in several euteleosts (Table 1). Like *scpp3* and *scpp5*, *scpplpq4* was identified in all teleosts except syngnathids. In zebrafish (*Danio rerio*), *scpplpq4* has a premature termination codon in the Tuebingen strain, but *scpplpq4* was apparently functional in other strains and other species of *Danio* (Fig. S3). Among teleosts, *scpplpq20* was identified in elopomorphs and an osteoglossomorph but not in otocephalans or euteleosts (Table 1).

In the NCBI database, *scpp7* shows an extremely high coverage of scale RNA-seq reads in sea bass (Table S1). Furthermore, no functional *scpp7* was identified in scaleless teleosts, like catfishes (Siluriformes) and sticklebacks (Fink and Fink 1981; Sire and Huysseune 2003). In cluster IIa, *scpp8* was found adjacent to *spp1* in most teleosts (Fig. S1). Adjacent to *scpp8*, both *scpp11a* and *scpp11b* were found in five otocephalans, including zebrafish and two euteleosts (Table 1). In all these species, *scpp12*, *scpp13*, and *scpp14* were found in cluster IIb. Although *scpp13* was solely found in denticle herring, no functional *scpp11a*, *scpp11b*, *scpp12*, *scpp13*, or *scpp14* was identified in any other teleosts. RNA-seq analysis showed that all these five genes were expressed in zebrafish skin (Table S2) (Thompson et al. 2021). For *gsp37*, a high coverage of scale RNA-seq reads was found in sea bass in the NCBI database (Table S1). Furthermore, *gsp37* was not found in scaleless teleosts, like catfishes and sticklebacks (Table 1).

No strongly expressed domains of teleost *scpplpq8* or *scpplpq5* genes were found in the NCBI database. Among teleosts, *scpplpq8* was identified only in five otocephalans, and *scpplpq5* was found in elopomorphs, an osteoglossomorph, and all otocephalans, but only in Salmoniformes among euteleosts (Table 1). To date, *scpp17* was found only in denticle herring and Mexican tetra. The ortholog of *scpp17* was not clearly identified in nonteleost actinopterygians, although *scpp17* and bowfin *scpplpq3* encode proteins showing weak sequence similarity.

## Discussion

### Acidic SCPP Genes and Bone in Teleosts

Among SCPP genes, *scpp1* and *spp1* are the only genes identified in all teleosts studied to date. In zebrafish, *scpp1* expression was detected mainly in odontoblasts and osteocytes, while the expression of *spp1* was found in osteoblasts (Kawasaki 2009). Unlike most noneuteleosts, bone is acellular (no osteocytes) in most euteleosts (Davesne et al. 2019). However, in pipefish among euteleosts, the expression of *scpp1* and *spp1* was detected in osteoblasts (Healey et al. 2024). The expression of *scpp1* and *spp1* in bone at least partly explains the presence of *scpp1* and *spp1* in syngnathids, which are toothless and scaleless but are encased in bony rings (Paulus 1999).

The ocean sunfish develops weakly ossified endoskeleton (Brown and Manzanares 2024). Nevertheless, both *scpp1* and *spp1* were apparently functional (Pan et al. 2016). Within the same Tetraodontiformes, boxfishes develop a carapace, which is composed of dermal plates, each consisting of the bone-like surface and lightly mineralized base (Yang et al. 2015). As expected, both *scpp1* and *spp1* are expressed during carapace formation (Hu et al. 2025). These studies illustrate that *scpp1* and *spp1* are employed widely for the mineralization of endoskeleton and exoskeleton.

In most euteleosts, mineral homeostasis seems to be less efficient because of the lack of osteocytes (Davesne et al. 2019), and some acidic SCPP genes involved in mineral homeostasis could be less important. Indeed, *ibsp* was found in all investigated noneuteleosts but only in chum salmon, northern pike, soldierfish, and wrasses among euteleosts (Table 1). In Salmoniformes, although salmons have cellular bone, pikes have acellular bone (Davesne et al. 2019). Moreover, the soldierfish and wrasse lineages retain *ibsp* long after cellular bone was lost in the common ancestor of euteleosts (Davesne et al. 2019). Thus, the phylogenetic distribution of *ibsp* and other acidic SCPP genes does not support a strong correlation between the evolution of acellular bone and the loss of specific acidic SCPP genes, although the lack of osteocytes may be one of many factors that are associated with the loss of some acidic SCPP genes.

### SCPP Gene Repertoire and Teeth in Teleosts

**Fig. 3.**
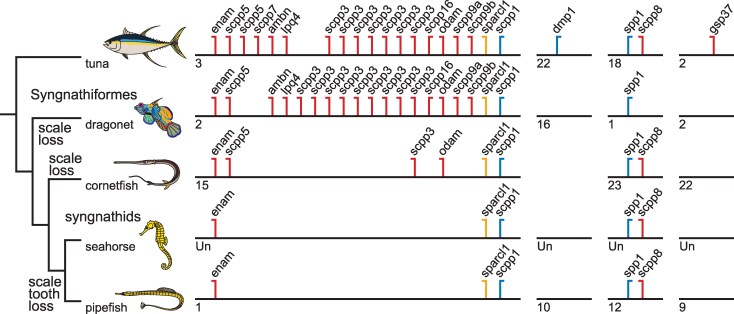
Chromosomal distribution of SCPP genes in yellowfin tuna and four Syngnathiformes species and the loss of scales and teeth. Each red, yellow, and blue inverted “L” represents the order and transcriptional direction of a P/Q-rich SCPP gene, *sparcl1*, and an acidic SCPP gene, respectively. Apparently nonfunctional tiger tail seahorse *scpp5* is not shown here. The distribution of these SCPP genes suggests that *scpp7* and *gsp37* were lost within Syngnathiformes in association with the loss of scales. In addition, *ambn*, *scpplpq4* (lpq4), *scpp16*, *scpp9a*, and *scpp9b* were lost before teeth were lost in the common ancestor of syngnathids, and functional *scpp5*, *scpp3*, and *odam* were lost concurrently with the loss of teeth. In euteleosts, *dmp1* was lost many times (Table 1). In cornetfish, cluster Ib was not thoroughly investigated because of limited gene annotations. The number and Un (unknown) represent the chromosome, where these clusters are located.

Previous studies identified only three functional SCPP genes, *enam*, *scpp1*, and *spp1*, in toothless and scaleless syngnathids and proposed that the loss of many SCPP genes, particularly *scpp5*, explains the loss of their teeth (Lin et al. 2016; Zhang et al. 2020b; Qu et al. 2021). In addition to these genes, *scpp8* was identified in the present study (Table 1). The expression of *scpp8* was highly upregulated in the anterior kidney by mycobacteriosis in seahorse (Table S1) (Fogelson et al. 2017), which may partly explain the presence of *scpp8* in syngnathids. Cornetfishes are phylogenetically close to syngnathids and are also scaleless, but grow teeth (Fig. 3) (Fritzsche and Thiesfeld 1999). A previous study found *odam* in cornetfish, in addition to SCPP genes found in syngnathids (Healey et al. 2024). In the present study, *scpp5* and *scpp3* were additionally identified in cornetfishes primarily by manual searches for protein-coding exons in the genome (Fig. 3, Fig. S4; see Materials and Methods). Significantly, *scpp5*, *scpp3*, and *odam* were identified in all investigated teleosts except syngnathids (Table 1). These findings imply that *scpp5*, *scpp3*, and *odam*, expressed during tooth formation in torafugu, zebrafish, or medaka (Kawasaki et al. 2005; Kawasaki 2009; Morita et al. 2023), have essential roles in tooth formation.

Among *scpp5*, *scpp3*, and *odam*, *scpp5* is expressed in both odontoblasts and the inner dental epithelium, *scpp3* is expressed in the outer dental epithelium, and *odam* is expressed in the inner dental epithelium (Kawasaki et al. 2005; Kawasaki 2009; Karagic et al. 2020; Rosa et al. 2021; Morita et al. 2023). The expression of *scpp5* is found during the formation of tooth cap enameloid, whereas the expression of *odam* begins after the cap enameloid matrix is formed (Kawasaki 2009). Furthermore, *scpp5* is expressed in odontoblasts but not in adjacent bone forming cells (Rosa et al. 2021). The distinct spatiotemporal expression pattern of *scpp5*, *scpp3*, and *odam* implies their specific roles, which supports the hypothesis that *scpp5*, *scpp3*, and *odam* play essential roles in tooth formation.

Within Syngnathiformes, mandarin dragonet is an outgroup of syngnathids and cornetfish, and it is also scaleless (Sadovy et al. 2005) but grows teeth, like cornetfish (Fig. 3). In mandarin dragonet, *ambn*, *scpp9a*, *scpp9b*, *scpp16*, and *scpplpq4* were found among SCPP genes missing in both syngnathids and cornetfish (Fig. 3). Notably, the expression of *ambn*, *scpp9a*, *scpp9b*, and *scpp16* was detected in teeth or suggested in tooth-bearing bone or tooth plates, as noted above. It is, thus, possible that the loss of these genes, and probably the loss of multicopy *scpp3* genes, in cornetfish is associated with the reduction or modification in some aspects of teeth. The phylogenetic distribution of these genes suggests that various SCPP genes were lost within Syngnathiformes immediately before and concurrently with the loss of teeth.

### SCPP Genes and Scale Reduction and Loss in Teleosts—Revisiting Previous Hypotheses

In most teleosts, the bulk of scales consists of the well mineralized external layer and the incompletely mineralized basal plate composed of elasmodin (Sire and Akimenko 2004). As both tissues are rich in collagen, like bone and dentin, *scpp1* and *spp1* are likely involved in mineralization of these scale tissues. Indeed, in zebrafish, the expression of both *scpp1* and *spp1* was upregulated during regeneration of scales, and a non-sense mutation in *spp1* resulted in small scales (Bergen et al. 2022).

Unlike the study of zebrafish *scpp1*, it was argued that defects in *scpp1* or *scpp5* would cause the loss of scales because one or both of these genes were missing or nonfunctional in scaleless teleosts, including Asian icefish (*Protosalanx chinensis*) (Zhang et al. 2020a), catfishes, electric eel, and three- stickleback (Liu et al. 2016). However, Asian icefish possesses scales (Roberts 1984), as discussed below. Moreover, a subsequent study identified apparently functional *scpp1* and *scpp5* in all these teleosts (Thompson et al. 2021).

In Mexican tetra, cave-dwelling populations grow smaller scales than surface-dwelling populations (Simon et al. 2017), and both *enam* and *scpp1* were described as nonfunctional in a cave-dwelling population (Lv et al. 2017). However, the present study identified apparently functional *enam* and *scpp1* (Fig. S5) and the identical repertoire of SCPP genes in cave-dwelling and surface-dwelling populations (Table S1). In various skin air-breathing fishes, like mudskippers, scales are reduced in size and number (Park 2002). A previous study found only nine SCPP genes in mudskippers and proposed that the loss of many SCPP genes could have led to their reduced scales (Bian et al. 2024). By contrast, the present study identified 19 SCPP genes in mudskippers (Table 1; Fig. S6), which does not support the association between scale reduction and loss of SCPP genes. In mudskippers, scales form layers and appreciably grow underneath the epidermal layer (Park 2002; Zhang et al. 2003). The reduction of scales is probably slight in mudskippers.

As described above, the present study identified many previously unrecognized SCPP genes. This is largely because earlier studies relied heavily on conventional sequence similarity searches, which are often ineffective in detecting SCPP genes that evolve rapidly (see Materials and Methods).

### SCPP Genes Vital to Scale Formation

In zebrafish, a strong expression of *enam* and *scpp7* was detected in various cells of epidermis and dermis, and a weaker expression of *ambn* was detected in a sub-population of basal epidermal cells that overlay scales (Aman et al. 2023). Furthermore, an upregulation of *scpp7*, *ambn*, *scpp5*, and *scpp8* expression was detected at the onset of scale formation (Zhang et al. 2022). However, during scale regeneration, while an upregulation of *scpp7* and *scpp5* expression was detected, the expression of *enam*, *ambn*, and *scpp8* was either not detected or not upregulated (Bergen et al. 2022). In addition, a high expression of *enam*, *scpp7*, and *scpp11a*, a slightly lower expression of *ambn*, *scpp11b*, *scpp13*, and *scpp14*, and a substantial expression of *gsp37* and *scpp12* were detected in the skin (Table S2) (Thompson et al. 2021). These results illustrate a vital role of *scpp7* in scale formation. By contrast, the significance of *enam*, *scpp5*, *ambn*, and *scpp8* in scale formation is equivocal. However, among five teleosts, in which *enam* was missing, apparently nonfunctional, or truncated, scales were reduced or lost in European eel, channel catfish, zig-zag eel, and Asian swamp eel (Table 1). Furthermore, among 11 scaleless teleosts, no functional *ambn* was identified in six species. Both *enam* and *ambn* appear to be important for some specific features of scales.

In addition to *scpp7*, *gsp37* is important for scale mineralization (Miyabe et al. 2012). In the present study, while no functional *scpp7* was identified, apparently functional *gsp37* was found in eight teleosts: European eel, elephant fish, pond loach, butterfly loach, northern pike, Atlantic cod, zig-zag eel, and mangrove killifish (Table 1). In European eel, scales are minute and embedded (Nelson et al. 2016). Both pond loach and mangrove killifish are prominent skin air-breathing teleosts, many of which are scaleless or develop reduced scales (Park 2002). Pond loach possesses thin and small scales deep in dermis (Park and Kim 1999), and scales of mangrove killifish are discontinuous (Wright 2012). Zig-zag eel also breathes air through the skin, and their thin scales are embedded in the dermis (Mittal and Munshi 1971). In loaches, including butterfly loach, scales are more or less degenerated (Cockerell 1909). It is likely that reduced scales are associated with the loss of functional *scpp7* in some of these teleosts. Small scales are also found in Atlantic cod (Ytteborg et al. 2020). However, it is not clear whether the absence of *scpp7* explains merely small scales of Atlantic cod. Moreover, no functional *scpp7* was identified in elephant fish or northern pike with no apparently reduced scales. Unexpectedly, *scpp7* appeared to be functional in a different pike, muskellunge (Fig. S7). These findings illustrate that the absence of *scpp7* is not fully associated with reduced scales in some teleosts.

Conversely, while *scpp7* was apparently functional, no functional *gsp37* was found in Asian icefish, sharksucker, frogfish, or torafugu (Table 1). In Asian icefish, fewer than 30 scales grow in males as a sexual organ only during the mating season (Roberts 1984; Saruwatari et al. 2002). Sharksucker possesses minute scales, usually embedded in the skin (Fischer and Bianchi 1984). Frogfish and fugu (*Takifugu*) grow spinules or s (Pietsch 1984; Shono et al. 2019). It was proposed that s of fugu evolved through the loss of the basal cycloid scale compartment (Shono et al. 2019). The basal compartment remains in ocean sunfish, which is phylogenetically close to fugu. In sunfish, both *scpp7* and *gsp37* were apparently functional. Thus, the loss of functional *gsp37* seems to be associated with the modification, reduction, or loss of scales in Asian icefish, sharksucker, frogfish, and fugu.

Both *scpp7* and *gsp37* were missing or apparently nonfunctional in 12 teleosts: stone loach (*Triplophysa dalaica*), electric eel, catfishes, mandarin dragonet, syngnathids, Asian swamp eel (Liem 1967), clingfish (Fricke et al. 2017), and sticklebacks (Table 1). All these teleosts are scaleless, except stone loach, in which the presence or absence of scales is unclear. However, both *scpp7* and *gsp37* were missing or apparently nonfunctional in *Triplophysa dosa* (Fig. S8), which was reported as scaleless (Chen and Yang 2005). Furthermore, while European eel has minute scales, phylogenetically close conger is scaleless (Nelson et al. 2016). In conger, neither *scpp7* nor *gsp37* was apparently functional (Fig. S9). The loss of scales in these teleosts appears to be associated with the loss of function in both *scpp7* and *gsp37*.

## Conclusions

Among all SCPP genes, only two acidic SCPP genes, *scpp1* and *spp1*, were identified in all investigated teleosts. Both *scpp1* and *spp1* are expressed during bone, tooth, and scale formation. However, the presence of *scpp1* and *spp1* in scaleless and toothless syngnathids underscores their significant roles in bone formation. Furthermore, investigation into the repertoire of SCPP genes in syngnathids and various scaleless teleosts suggests an essential contribution of *scpp5*, *scpp3*, and *odam* to tooth formation and *scpp7* and *gsp37* to scale formation.

These results reveal the basic repertoire of teleost SCPP genes for mineralized skeletal elements: *scpp1*, *spp1*, *scpp5*, *scpp3*, *odam*, *scpp7*, and *gsp37*. Among these seven SCPP genes, *spp1* and *odam* are shared with sarcopterygians including coelacanth, while the orthologs of the other five have remained unidentified in sarcopterygians to date.

Other than the basic repertoire, *ambn*, *scpp9a*, *scpp9b*, and *scpp16* appear to be associated with some aspects of teeth, while both *enam* and *ambn* are likely important for some specific features of scales. Nevertheless, the loss of SCPP genes does not necessarily associate with overt changes in skeletal tissues, as illustrated by pike *scpp7* genes. Thus, I suggest that the repertoire of SCPP genes in teleost species underlies diverse conditions of mineralized skeletal elements.

## Materials and Methods

In the present study, 63 teleost species (Fig. 1) were selected to investigate because the genome assembly was relatively well annotated and massive RNA-seq data were available. Initially, SCPP gene clusters were identified by finding genes located adjacent to known SCPP gene clusters in other species using BLAST at NCBI (https://www.ncbi.nlm.nih.gov/) (Altschul et al. 1990). Subsequently, SCPP genes were sought among annotated genes and RNA-seq exon coverage reads in SCPP gene clusters using the genome data viewer at NCBI. In genomic regions between annotated genes and RNA-seq exon coverage, SCPP genes were searched using gene prediction program Genscan (https://pbil.univ-lyon1.fr/members/duret/cours/INSA/exercise4/pgscan.html) (Burge and Karlin 1997). In this process, missing genes and exons were manually sought by looking for potential “AG” splice acceptor sites (Ma et al. 2015) and by translating the nucleotide sequence downstream of AG. SCPP genes between annotated genes and RNA-seq exon coverage were also searched by amino acid or nucleotide sequence similarities with BLAST using known SCPP gene sequences as queries. For genes that contain a premature termination codon, frameshift, noncanonical splice acceptor–donor sequence, or unusual protein-coding sequence, the genome sequence was reassembled by BLASTN using the Sequence Read Archive (SRA) data. Raw SRA data were assembled using CAP3 (Huang and Madan 1999) at Pole Rhone-Alpes de Bioinformatique Site Doua (https://doua.prabi.fr/software/cap3). Diagnosis and characteristics of each SCPP gene are summarized in Supplementary Notes. Table S1 shows genome sequence assemblies, RNA-seq data, and SRA data used in this study and gene ID, genomic coordinates of each SCPP gene, and corrections of genome assemblies made in this study.

Many previous studies have failed to identify certain SCPP genes that are critical for supporting their hypotheses. This is mainly because those studies relied heavily on conventional sequence similarity searches sometimes using sequences of orthologs in phylogenetically distant species as queries. In such analyses, it is difficult to identify SCPP genes that lack evolutionarily conserved modular structures and are susceptible to extensive amino acid substitutions, intraexonic duplications and deletions, and exon duplications and deletions (Chun et al. 2025). As noted above, the present study used massive RNA-seq data and manual procedures, which significantly contributed to identifying many previously unrecognized SCPP genes. A deep knowledge about the characteristics of SCPP genes (Kawasaki and Weiss 2003, 2006) is also essential for the identification of SCPP genes.

## Supplementary Material

evag148_Supplementary_Data

## Data Availability

All data used in this study are summarized in Table S1 or Supplementary Information.
