# Supplementary material for: SCPP Gene Repertoires in Teleosts and Evolutionary Changes in Bone, Teeth, and Scales
Source: Genome Biol Evol. 2026 Jun 22;18(7):evag148. doi: 10.1093/gbe/evag148 (PMC13367325; doi:10.1093/gbe/evag148)
Supplement: evag148_Supplementary_Data [file evag148_supplementary_data.zip › SupplMaterial2.pdf]

Supplementary Information for SCPP Gene Repertoires in Teleosts and Evolutionary Changes in Bone, Teeth, and Scales.

Fig. S1 The arrangement of SCPP genes in 63 teleost species. pages 2-5
Fig. S2 Similarities between *scpp12* and *scpplpq12* and between *scpp13*, *scpp14*, and *scpplpq9*. page 6
Fig. S3 Zebrafish *scpplpq4*. page 7
Fig. S4 Amino acid sequences encoded by *scpp5* and *scpp3* in cornetfish. Page 8
Fig. S5 Amino acid sequences encoded by *enam* and *scpp1* in surface dwelling and Tinaja cave dwelling Mexican tetra. page 9
Fig. S6 SCPP genes in mudskippers. page 10
Fig. S7 Pike *scpp7* genes. page 11
Fig. S8 Triplophysa *gsp37* genes. page 12
Fig. S9 Conger *gsp37* and *scpp7*. page 13

Supplementary Notes.
Genes Encoding P/Q-rich SCPP Genes pages 14-18
Genes Encoding Acidic SCPP Genes pages 18-19

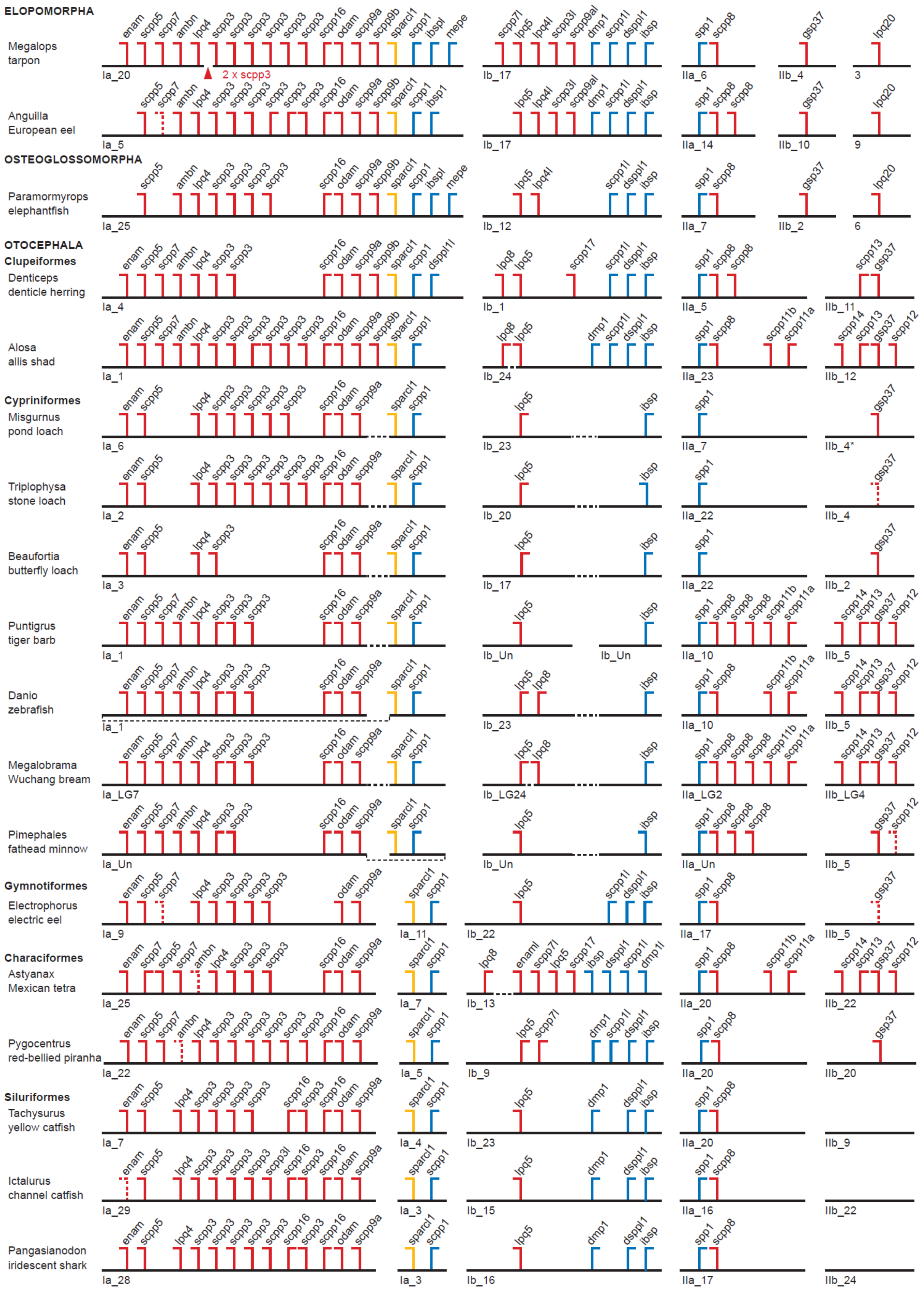
ELOPOMORPHA
Megalops tarpon
enam scpp5 scpp7 ambn lpq4 scpp3 scpp3 scpp3 scpp3 scpp3 scpp3 scpp16 odam scpp9a scpp9b sparcl1 scpp1 ibspl mepe
Ia_20
2 x scpp3
scpp7l lpq5 lpq4l scpp3l scpp9al dmp1 scpp1l dspp1l ibsp
Ib_17
spp1 scpp8
IIa_6
gsp37
IIb_4
lpq20
3
Anguilla European eel
scpp5 scpp7 ambn lpq4 scpp3 scpp3 scpp3 scpp3 scpp3 scpp3 scpp16 odam scpp9a scpp9b sparcl1 scpp1 ibspl
Ia_5
lpq5 lpq4l scpp3l scpp9al dmp1 scpp1l dspp1l ibsp
Ib_17
spp1 scpp8 scpp8
IIa_14
gsp37
IIb_10
lpq20
9
OSTEOGLOSSOMORPHA
Paramormyrops elephantfish
scpp5 ambn lpq4 scpp3 scpp3 scpp3 scpp3 scpp16 odam scpp9a scpp9b sparcl1 scpp1 ibspl mepe
Ia_25
lpq5 lpq4l scpp1l dspp1l ibsp
Ib_12
spp1 scpp8
IIa_7
gsp37
IIb_2
lpq20
6
OTOCEPHALA
Clupeiformes
Denticeps denticle herring
enam scpp5 scpp7 ambn lpq4 scpp3 scpp3 scpp16 odam scpp9a scpp9b sparcl1 scpp1 dspp1l
Ia_4
lpq8 lpq5 scpp17 scpp1l dspp1l ibsp
Ib_1
spp1 scpp8 scpp8
IIa_5
scpp13 gsp37
IIb_11
Alosa allis shad
enam scpp5 scpp7 ambn lpq4 scpp3 scpp3 scpp3 scpp3 scpp3 scpp3 scpp16 odam scpp9a scpp9b sparcl1 scpp1
Ia_1
lpq8 lpq5 dmp1 scpp1l dspp1l ibsp
Ib_24
spp1 scpp8 scpp11b scpp11a
IIa_23
scpp14 scpp13 gsp37 scpp12
IIb_12
Cypriniformes
Misgurnus pond loach
enam scpp5 lpq4 scpp3 scpp3 scpp3 scpp3 scpp3 scpp16 odam scpp9a sparcl1 scpp1
Ia_6
lpq5 ibsp
Ib_23
spp1
IIa_7
gsp37
IIb_4*
Triplophysa stone loach
enam scpp5 lpq4 scpp3 scpp3 scpp3 scpp3 scpp3 scpp3 scpp16 odam scpp9a sparcl1 scpp1
Ia_2
lpq5 ibsp
Ib_20
spp1
IIa_22
gsp37
IIb_4
Beaufortia butterfly loach
enam scpp5 lpq4 scpp3 scpp16 odam scpp9a sparcl1 scpp1
Ia_3
lpq5 ibsp
Ib_17
spp1
IIa_22
gsp37
IIb_2
Puntigrus tiger barb
enam scpp5 scpp7 ambn lpq4 scpp3 scpp3 scpp3 scpp16 odam scpp9a sparcl1 scpp1
Ia_1
lpq5 ibsp
Ib_Un Ib_Un
spp1 scpp8 scpp8 scpp8 scpp11b scpp11a
IIa_10
scpp14 scpp13 gsp37 scpp12
IIb_5
Danio zebrafish
enam scpp5 scpp7 ambn lpq4 scpp3 scpp3 scpp3 scpp16 odam scpp9a sparcl1 scpp1
Ia_1
lpq5 lpq8 ibsp
Ib_23
spp1 scpp8 scpp11b scpp11a
IIa_10
scpp14 scpp13 gsp37 scpp12
IIb_5
Megalobrama Wuchang bream
enam scpp5 scpp7 ambn lpq4 scpp3 scpp3 scpp3 scpp16 odam scpp9a sparcl1 scpp1
Ia_LG7
lpq5 lpq8 ibsp
Ib_LG24
spp1 scpp8 scpp8 scpp8 scpp11b scpp11a
IIa_LG2
scpp14 scpp13 gsp37 scpp12
IIb_LG4
Pimephales fathead minnow
enam scpp5 scpp7 ambn lpq4 scpp3 scpp3 scpp16 odam scpp9a sparcl1 scpp1
Ia_Un
lpq5 ibsp
Ib_Un
spp1 scpp8 scpp8 scpp8
IIa_Un
gsp37 scpp12
IIb_5
Gymnotiformes
Electrophorus electric eel
enam scpp5 scpp7 lpq4 scpp3 scpp3 scpp3 scpp3 odam scpp9a
Ia_9
sparcl1 scpp1
Ia_11
lpq5 scpp1l dspp1l ibsp
Ib_22
spp1 scpp8
IIa_17
gsp37
IIb_5
Characiformes
Astyanax Mexican tetra
enam scpp7 scpp5 scpp7 ambn lpq4 scpp3 scpp3 scpp3 scpp16 odam scpp9a
Ia_25
sparcl1 scpp1
Ia_7
lpq8 enaml scpp7l lpq5 scpp17 ibsp dspp1l scpp1l dmp1l
Ib_13
spp1 scpp8 scpp11b scpp11a
IIa_20
scpp14 scpp13 gsp37 scpp12
IIb_22
Pygocentrus red-bellied piranha
enam scpp5 scpp7 ambn lpq4 scpp3 scpp3 scpp3 scpp3 scpp3 scpp3 scpp16 odam scpp9a
Ia_22
sparcl1 scpp1
Ia_5
lpq5 scpp7l dmp1 scpp1l dspp1l ibsp
Ib_9
spp1 scpp8
IIa_20
gsp37
IIb_20
Siluriformes
Tachysurus yellow catfish
enam scpp5 lpq4 scpp3 scpp3 scpp3 scpp3 scpp16 scpp3 scpp16 odam scpp9a
Ia_7
sparcl1 scpp1
Ia_4
lpq5 dmp1 dspp1l ibsp
Ib_23
spp1 scpp8
IIa_20
IIb_9
Ictalurus channel catfish
enam scpp5 lpq4 scpp3 scpp3 scpp3 scpp3 scpp3l scpp16 scpp3 scpp16 odam scpp9a
Ia_29
sparcl1 scpp1
Ia_3
lpq5 dmp1 dspp1l ibsp
Ib_15
spp1 scpp8
IIa_16
IIb_22
Pangasianodon iridescent shark
enam scpp5 lpq4 scpp3 scpp3 scpp3 scpp3 scpp3 scpp16 scpp3 scpp16 odam scpp9a
Ia_28
sparcl1 scpp1
Ia_3
lpq5 dmp1 dspp1l ibsp
Ib_16
spp1 scpp8
IIa_17
IIb_24

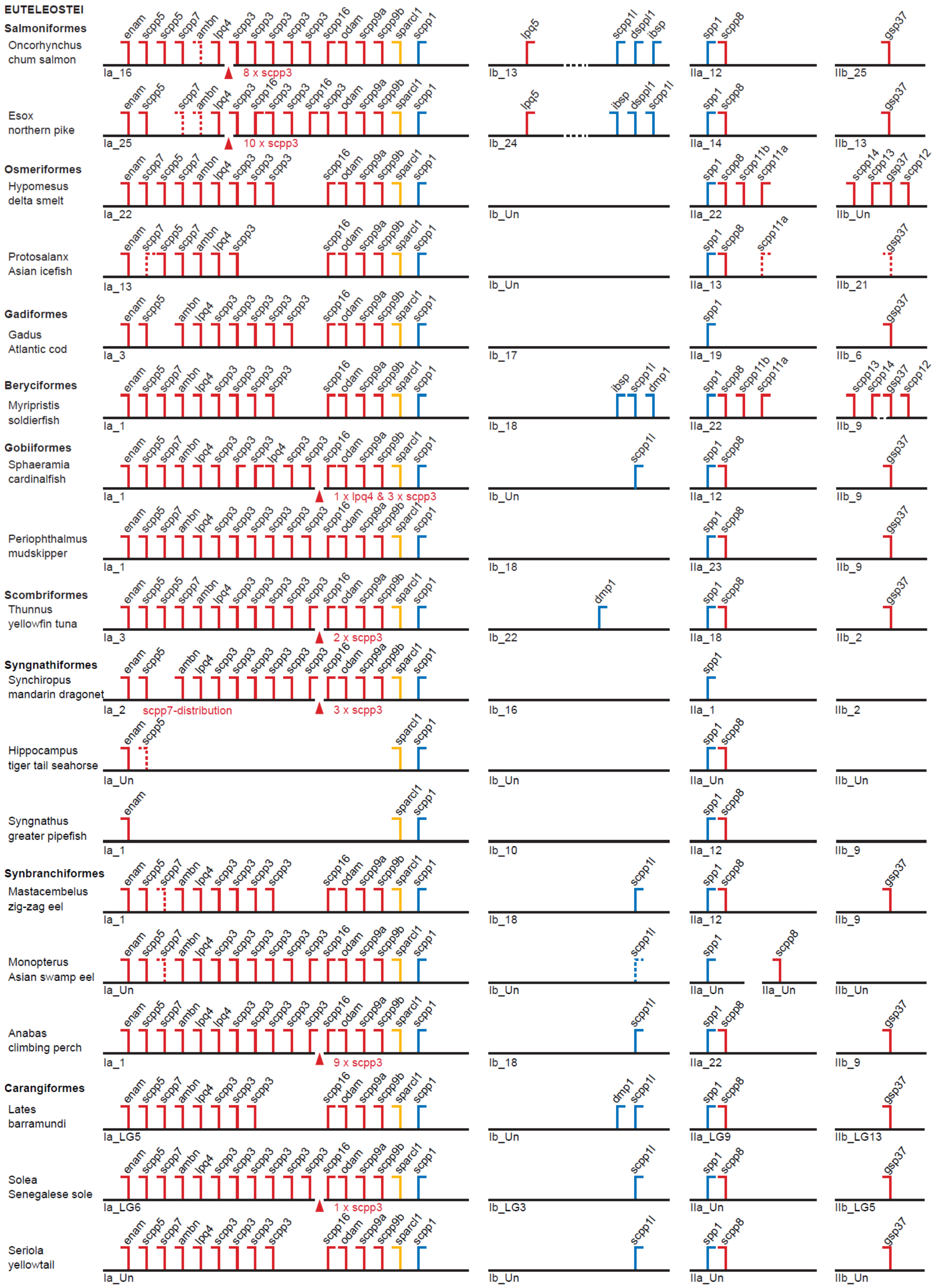
EUTELEOSTEI
Salmoniformes
Oncorhynchus chum salmon
enam scpp5 scpp5 scpp7 ambn lpq4 scpp3 scpp3 scpp3 scpp3 scpp3 scpp16 odam scpp9a scpp9b sparcl1 scpp1
Ia_16
8 x scpp3
lpq5 scpp1l dspp1l ibsp
Ib_13
spp1 scpp8
IIa_12
gsp37
IIb_25
Esox northern pike
enam scpp5 scpp7 ambn lpq4 scpp3 scpp16 scpp3 scpp3 scpp16 scpp3 odam scpp9a scpp9b sparcl1 scpp1
Ia_25
10 x scpp3
lpq5 ibsp dspp1l scpp1l
Ib_24
spp1 scpp8
IIa_14
gsp37
IIb_13
Osmeriformes
Hypomesus delta smelt
enam scpp7 scpp5 scpp7 ambn scpp3 scpp3 scpp3 scpp16 odam scpp9a scpp9b sparcl1 scpp1
Ia_22
Ib_Un
spp1 scpp8 scpp11b scpp11a
IIa_22
scpp14 scpp13 gsp37 scpp12
IIb_Un
Protosalanx Asian icefish
enam scpp7 scpp5 scpp7 ambn lpq4 scpp3 scpp16 odam scpp9a scpp9b sparcl1 scpp1
Ia_13
Ib_Un
spp1 scpp8 scpp11a
IIa_13
gsp37
IIb_21
Gadiformes
Gadus Atlantic cod
enam scpp5 ambn lpq4 scpp3 scpp3 scpp3 scpp3 scpp3 scpp16 odam scpp9a scpp9b sparcl1 scpp1
Ia_3
Ib_17
spp1
IIa_19
gsp37
IIb_6
Beryciformes
Myripristis soldierfish
enam scpp5 scpp7 ambn lpq4 scpp3 scpp3 scpp3 scpp3 scpp16 odam scpp9a scpp9b sparcl1 scpp1
Ia_1
ibsp scpp1l dmp1
Ib_18
spp1 scpp8 scpp11b scpp11a
IIa_22
scpp13 scpp14 gsp37 scpp12
IIb_9
Gobiiformes
Sphaeramia cardinalfish
enam scpp5 scpp7 ambn lpq4 scpp3 scpp3 scpp3 lpq4 scpp3 scpp3 scpp16 odam scpp9a scpp9b sparcl1 scpp1
Ia_1
1 x lpq4 & 3 x scpp3
scpp1l
Ib_Un
spp1 scpp8
IIa_12
gsp37
IIb_9
Periophthalmus mudskipper
enam scpp5 scpp7 ambn lpq4 scpp3 scpp3 scpp3 scpp3 scpp3 scpp3 scpp16 odam scpp9a scpp9b sparcl1 scpp1
Ia_1
Ib_18
spp1 scpp8
IIa_23
gsp37
IIb_9
Scombriformes
Thunnus yellowfin tuna
enam scpp5 scpp5 scpp7 ambn lpq4 scpp3 scpp3 scpp3 scpp3 scpp3 scpp16 odam scpp9a scpp9b sparcl1 scpp1
Ia_3
2 x scpp3
dmp1
Ib_22
spp1 scpp8
IIa_18
gsp37
IIb_2
Syngnathiformes
Synchiropus mandarin dragonet
enam scpp5 ambn lpq4 scpp3 scpp3 scpp3 scpp3 scpp3 scpp16 odam scpp9a scpp9b sparcl1 scpp1
Ia_2
scpp7-distribution
3 x scpp3
Ib_16
spp1
IIa_1
IIb_2
Hippocampus tiger tail seahorse
enam scpp5 sparcl1 scpp1
Ia_Un
Ib_Un
spp1 scpp8
IIa_Un
IIb_Un
Syngnathus greater pipefish
enam sparcl1 scpp1
Ia_1
Ib_10
spp1 scpp8
IIa_12
IIb_9
Synbranchiformes
Mastacembelus zig-zag eel
enam scpp5 scpp7 ambn lpq4 scpp3 scpp3 scpp3 scpp3 scpp16 odam scpp9a scpp9b sparcl1 scpp1
Ia_1
scpp1l
Ib_18
spp1 scpp8
IIa_12
gsp37
IIb_9
Monopterus Asian swamp eel
enam scpp5 scpp7 ambn lpq4 scpp3 scpp3 scpp3 scpp3 scpp3 scpp16 odam scpp9a scpp9b sparcl1 scpp1
Ia_Un
scpp1l
Ib_Un
spp1
IIa_Un
scpp8
IIa_Un
IIb_Un
Anabas climbing perch
enam scpp5 scpp7 ambn lpq4 scpp3 scpp3 scpp3 scpp3 scpp3 scpp16 odam scpp9a scpp9b sparcl1 scpp1
Ia_1
9 x scpp3
scpp1l
Ib_18
spp1 scpp8
IIa_22
gsp37
IIb_9
Carangiformes
Lates barramundi
enam scpp5 scpp7 ambn lpq4 scpp3 scpp3 scpp3 scpp16 odam scpp9a scpp9b sparcl1 scpp1
Ia_LG5
dmp1 scpp1l
Ib_Un
spp1 scpp8
IIa_LG9
gsp37
IIb_LG13
Solea Senegalese sole
enam scpp5 scpp7 ambn lpq4 scpp3 scpp3 scpp3 scpp3 scpp3 scpp16 odam scpp9a scpp9b sparcl1 scpp1
Ia_LG6
1 x scpp3
scpp1l
Ib_LG3
spp1 scpp8
IIa_Un
gsp37
IIb_LG5
Seriola yellowtail
enam scpp5 scpp7 ambn lpq4 scpp3 scpp3 scpp3 scpp16 odam scpp9a scpp9b sparcl1 scpp1
Ia_Un
scpp1l
Ib_Un
spp1 scpp8
IIa_Un
gsp37
IIb_Un

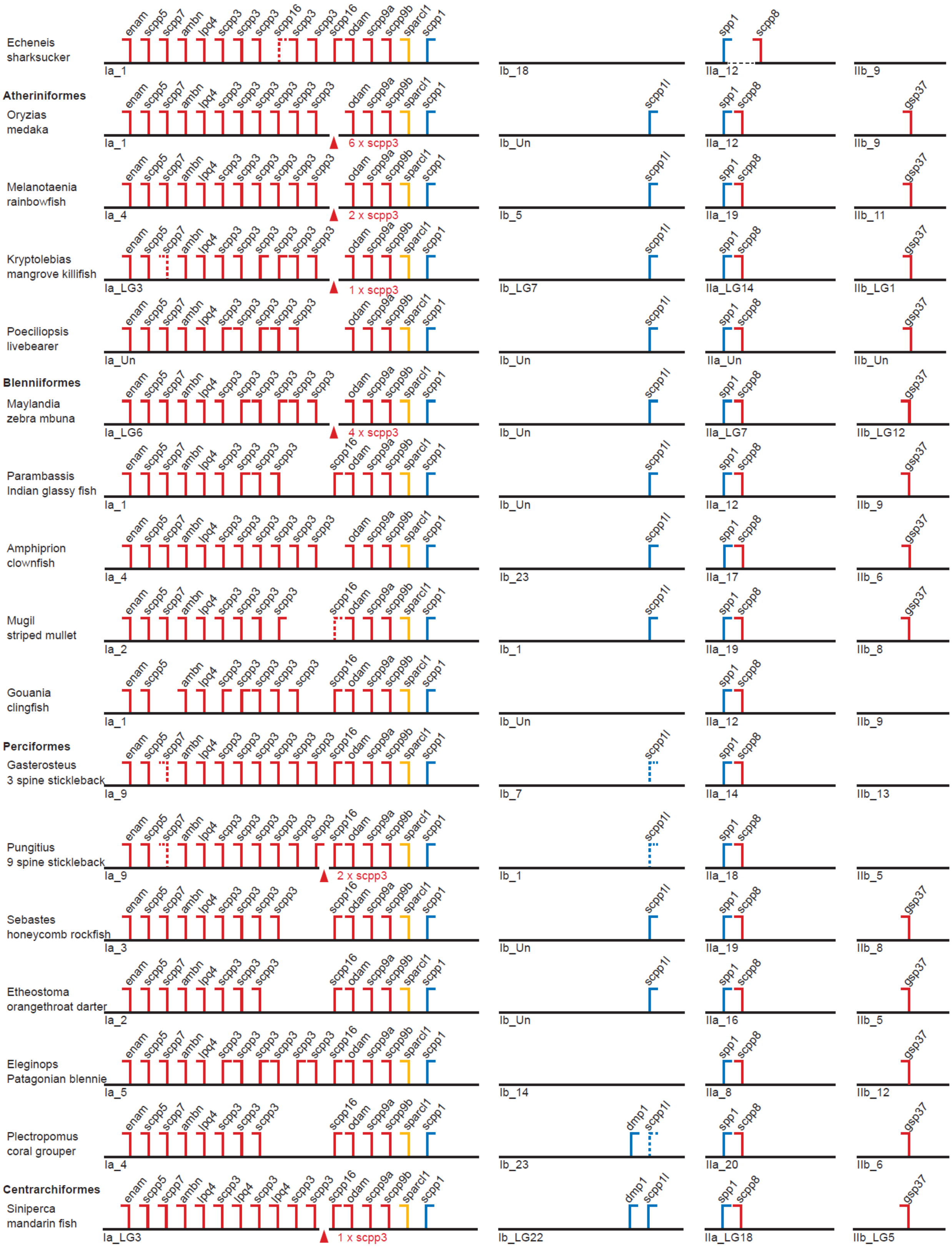
Echeneis
sharksucker
Ia_1
Ib_18
IIa_12
IIb_9
Atheriniformes
Oryzias
medaka
Ia_1
6 x scpp3
Ib_Un
IIa_12
IIb_9
Melanotaenia
rainbowfish
Ia_4
2 x scpp3
Ib_5
IIa_19
IIb_11
Kryptolebias
mangrove killifish
Ia_LG3
1 x scpp3
Ib_LG7
IIa_LG14
IIb_LG1
Poeciliopsis
livebearer
Ia_Un
Ib_Un
IIa_Un
IIb_Un
Blenniiformes
Maylandia
zebra mbuna
Ia_LG6
4 x scpp3
Ib_Un
IIa_LG7
IIb_LG12
Parambassis
Indian glassy fish
Ia_1
Ib_Un
IIa_12
IIb_9
Amphiprion
clownfish
Ia_4
Ib_23
IIa_17
IIb_6
Mugil
striped mullet
Ia_2
Ib_1
IIa_19
IIb_8
Gouania
clingfish
Ia_1
Ib_Un
IIa_12
IIb_9
Perciformes
Gasterosteus
3 spine stickleback
Ia_9
Ib_7
IIa_14
IIb_13
Pungitius
9 spine stickleback
Ia_9
2 x scpp3
Ib_1
IIa_18
IIb_5
Sebastes
honeycomb rockfish
Ia_3
Ib_Un
IIa_19
IIb_8
Etheostoma
orangethroat darter
Ia_2
Ib_Un
IIa_16
IIb_5
Eleginops
Patagonian blennie
Ia_5
Ib_14
IIa_8
IIb_12
Plectropomus
coral grouper
Ia_4
Ib_23
IIa_20
IIb_6
Centrarchiformes
Siniperca
mandarin fish
Ia_LG3
1 x scpp3
Ib_LG22
IIa_LG18
IIb_LG5
enam
scpp5
scpp7
ambn
lpq4
scpp3
scpp16
odam
scpp9a
scpp9b
sparcl1
scpp1
scpp1l
dmp1
spp1
scpp8
gsp37

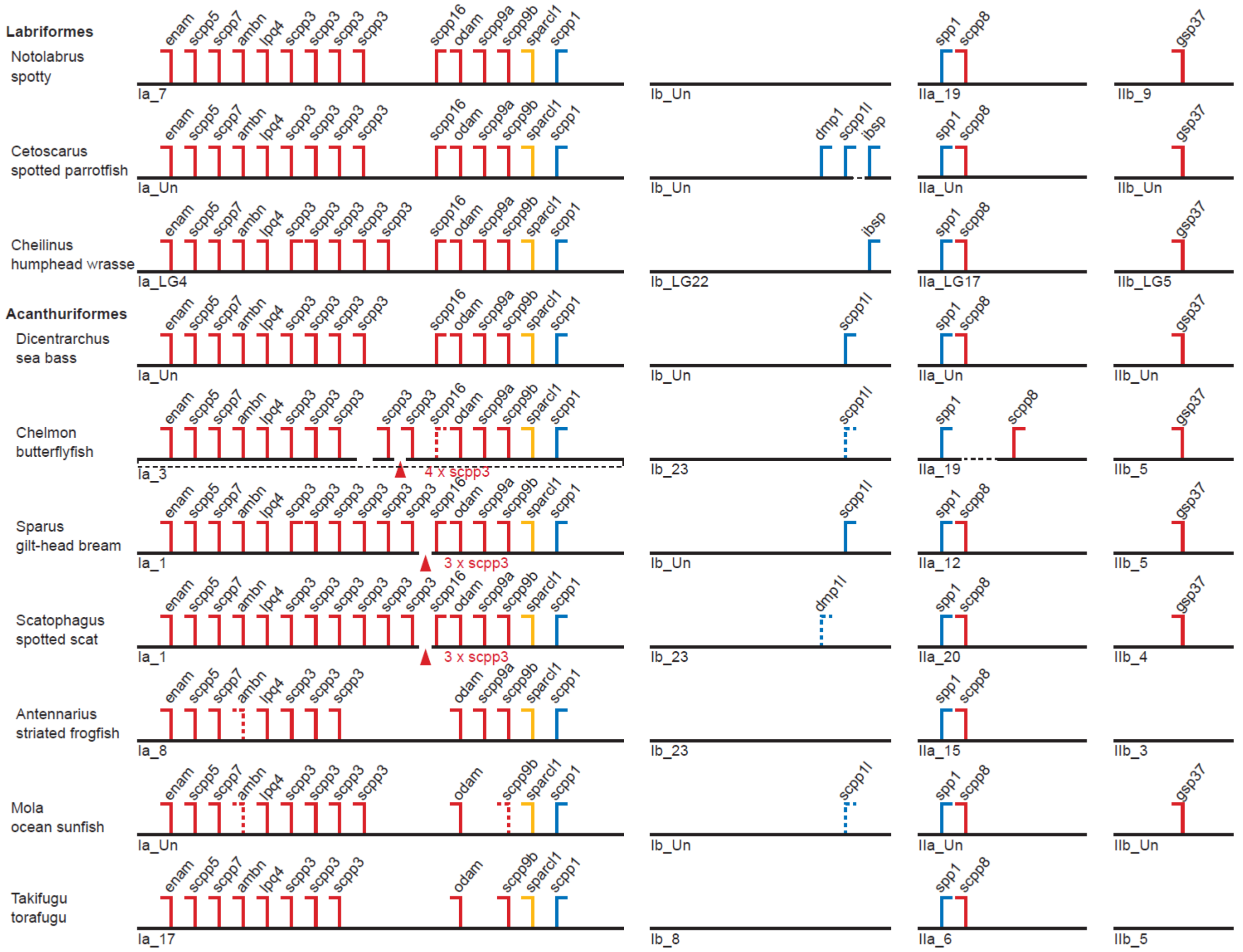

Fig. S1. The arrangement of SCPP genes in 63 teleost species. Each red, yellow, and blue inverted "L" represents the order and transcriptional direction of a P/Q-rich SCPP gene, *sparcl1*, and an acidic SCPP gene, respectively. An apparently functional gene is shown by a solid line, while non-functional gene is shown by a dashed line. Each horizontal line represents a chromosomal region. Two chromosomal regions separated by an intrachromosomal rearrangement are connected by a dashed horizontal line. The name of the gene is shown on the top of each inverted L, and the location of each cluster (Ia, Ib, IIa, and IIb) is shown below the horizontal line after "_" (number, chromosome; Un, unknown chromosome; or LG, linkage group). Gene names, lpq4, lpq5, and lpq8 represent *scpplpq4*, *scpplpq5*, and *scpplpq8*, respectively. Genus (top) and the common name (bottom) are shown on the left. In some species, a red triangle shows the presence of one or more *scpp3* or *scpplpq4* genes in the location.

```
(a)
scpp12
allis shad     GKAPGPGPSTQP-----TRAQPSFSSGPTIIKSGSKPIPPDGRQPPVIMFPGIVIP--ADIDIASILTQLLSQN
soldierfish    AKRIGIGESKGD-----VSAIGIGEGKKEVSGTNPGDDTSGGGESPLIIFPGVLIPDHEDSKTAADVQKLMIQN
scpplpq12      ..  *:**:*       .:*    *..: ***:*     : .* . ::****:::*  *.::*. ::   : ::
spotted gar    VGTGGMGETKPTPLVPATAATPTKESTQPVSGSN-----AESGPPNILIFPGIFLPIPEKTNTPENMVAALKKD
alligator gar  VGTGGMGETKPTPLVPATAATPTKESTQPVSGSN-----AESGPPNILIFPGIFLPIPEKTNTPENMVAALKKD

(b)
scpp13        pcexon1               pcexon2             pcexon3
d. herring    MLNAFALILGSLCLGL--AAP ADQQMG----PFSEGQ--- GVRVLLNKIRETRASINRPAHQQDAPLTWKEGRRVMPAAA
allis shad    MKAITLLL-GSLCVCL--AAP VEEHHERVARSALSGSEET GLRTLLTEVRDTLANRNQNPIAPQQPAPGITIQEPPPPMN
tiger barb    MRTFVLII-SNFFLIS--AAP VDQHHGIEARSVSEEG--- GLQDLITQMRDTLANVNKPPIAEPTTRPIGIQVQEPSPQL
zebrafish     MKITVLLI-SNLLLIT--AAP VDQHHEIEARSASEEDGQG ALQALLTEMRDTLANVNKPPITNPTTKPTGLQELPPQVQT
W. bream      MKTIVLII-SNLFLIT--AAP VDQNHDIEARSASEEE--- GLQALITQMRDTLANVNKPPIADPTPKPAGLQLQEPSSQF
delta smelt   MKLLVFLL-GLLITCH--TAP VEDEHRREARSASEEE--- VENLRLMLTEMKQTLAKVNQLPPAQPVASPKDPSIVVHSI
soldierfish   MIVQLFVL-SLIAACH--SAP VENR-KREARAISVEE--- QLFGLASVSREVGPIRIFPPLRPPPPLPPRVMPPTVFFNP
M. tetra      MRSAVFMF-SCLCLSL--AAP VEEHHEREARDASEEE--- DLRVLLTEMKETLATINNNNAPVPEVPPQLPQDPQASLQP
scpplpq9      *:  .:::   :  .*  *** *:  *:*:.*. * *
allig. gar    MKNIIMVL-GLLSSCL--AAP VNQMHERKARSISYE---- DFGFMMPRMMQTFSRYPFPIPFPVPRFLISLPSQPLPPPI
spotted gar   MKNIILVL-GLLSSCL--AAP VNQMHERKARSISYE---- DFGFMMPRMMQTFSRYPFPIPFPVPRFQISLPSQPLPPPI
reedfish      MKVIFLLL-LYVGFSL--AAP VDLKHKRKVRSISSE---- EFNYGYRWPRPSFIRYPYPFLVPIFPNYVPSQPAPVPEPM
S. bichir     MKVIFLLL-LYVGFSL--AAP VDLKHERKVRSISSE---- EFYYGYRRPRLSFIRYPYPFLVPIFPNNVPSQPAPAPEPM
scpp14        ** *:::*   .  .*  *** *.  *.**.*::: *
soldierfish  MMKTIIFML-GSFCLCL--AAP VHDAHDRKARALALE---- YQTASFEDGFRMPVFSHYPGFFPNFVNPGMFQNPFQPPVQ
delta smelt   MKTFILML-GLLYLCL--AAP MEQTEDRKIQLPSDV---- VPGSLDLPPQTAQPQQDGTPAFQPGLFPDPQMDHFLNPGV
M. tetra      MRSVTLLL-GLLSVVL--CAP AGR-RG--PSNLSVE---- DLLRVLPVPVPIPPVPVPPVPVAPVPLPPAQPVIYPEIYD
W. bream      MTTLILVFSLLSAVCLSLAVP VEKTRGRKTRDLSFE---- DFYRPVFPVQTMPSFPFLMNMDQFRGLQLTPYRFPSIVRP
zebrafish     MATLIFVYYLLTAVCLNLAAP VKETRSRRTRDMSIE---- DLYQPMRPGQSGFPFVFLMPTDQFPGMQLTPNISPSIIRP
tiger barb    MTTLILGFSLLSAACLSLAVP AEKT-SRKTRDLSFE---- EFYRPGLPVQSAPSVYFLVPVGQYGGQQLTPYRFPSLLRP
allis shad    MRVLLFTL-GLLGLGL--SAP VGPFDNN----LSAE---- DLLLQYLMQSNNVNPNPTYRFNISPYQNPMYGFNMFSFQQ
```

Fig. S2. Similarities between *scpp12* and *scpplpq12* (top) and between *scpp13*, *scpp14*, and *scpplpq9* (bottom). (**a**) Similarities in amino acid sequence near the C-terminal encode by allis shad and soldierfish *scpp12* genes and spotted gar and alligator gar *scpplpq12* genes. All these genes comprise two protein-coding exons (pcexons). "*" denotes identical amino acids, while ":" and "." represent strongly similar and weakly similar amino acids encoded by soldierfish *scpp12* and by spotted gar and alligator *scpplpq12* genes (expected value $2e^{-6}$). (**b**) All these genes comprise three protein-coding exons (pcexons). Amino acid sequences containing potentially phosphorylated Ser residues (pSer-Xaa-Glu; Xaa represents any amino acids) are shown in blue. Identical and similar amino acids encoded by pcexons 1 and 2 of Mexican tetra *scpp13* and gar and bichir *scpplpq9* genes, and by pcexons 1 and 2 of gar and bichir *scpplpq9* genes and soldierfish *scpp14* are shown as stated above. Similar sequences (FSR/HYP) encoded by pcexon 3 are underlined. Abbreviations: W. bream, Wuchang bream; M. tetra, Mexican tetra, S. bichir, Senegal bichir.

```
pcexon2                       L  .  H  R  Y  K  P  G  S  Y  S  G  S  D  E
JBMGRA010000001 TAATTCTCACAGCTCTGACACCGTTATAAGCCCGGGAGCTACTCTGGCAGTGATGAGGTAAACTCCCAGTG Tuebingen
CABZ01020235    TAATTCTCACAGCTCTGACACCGTTATAAGCCCGGGAGCTACTCTGGCAGTGATGAGGTAAACTCCCAGTG Tuebingen
JAHVXS010000001 TAATTCTCACAGCTCTGACACCGTTATAAGCCCGGGAGCTACTCTGGCAGTGATGAGGTAAACTCCCAGTG AB
QQSU01000001    TAATTCTCACAGCTCTGCCACCGTTATAAGCCCGGGAGCTACTCTGGCAGTGATGAGGTAAACTCCCAGTG AB
JALCZT010090672 TAATTCTCACAGCTCTGCCACCGTTATAAGCCCGGGAGCTACTCTGGCAGTGATGAGGTAAACTCCCAGTG AB3
JALCZT010090680 TAATTCTCACAGCTCTGCCACCGTTATAAGCCCGGGAGCTACTCTGGCAGTGATGAGGTAAACTCCCAGTG AB3
JALCZS010003720 TAATTCTCACAGCTCTGCCACCGTTATAAGCCCGGGAGCTACTCTGGCAGTGATGAGGTAAACTCCCAGTG CG1
LKPD02012851    TAATTCTCACAGCTCTGCCACCGTTATAAGCCCGGGAGCTACTCTGGCAGTGATGAGGTAAACTCCCAGTG CG2
CALUEL010000207 TAATTCTCACAGCTCTGCCACCGTTATAAGCCCGGGAGCTACTCTGGCAGTGATGAGGTAAACTCCCAGTG SAT
JAIHOL010000484 TAATTCTCACAGCTCTGCCACCGTTATAAGCCCGGGAGCTACTCTGGCAGTGATGAGGTAAACTCCCAGTG SAT
JAIHOL010000647 TAATTCTCACAGCTCTGCCACCGTTATAAGCCCGGGAGCTACTCTGGCAGTGATGAGGTAAACTCCCAGTG SAT
BTXV01000001    TAATTCTCACAGCTCTGCCACCGTTATAAGCCCGGGAGCTACTCTGGCAGTGATGAGGTAAACTCCCAGTG NIES-R
D aesculapii    AATTCCTCACAGCTCTGCCACCGTTATAAGCCCGGGAGCTACTCTGGCAGTGATGAGGTAAACTCCCAGTG
D albolineatus  AATTCCTCACAGCTCTGCCACCCTTATAAGCCCGGGAGCTACTCTGGCAGTGATGAGGTAAACTCCCAGTG
D choprai       AATTCCTCACAGCTCTGCCACCGTTATAAGCCCGGCAGCTACTCTGGCAGTGATGAGGTAAACTCCCAGTG
D jaintianensis AATTCCTCACAGCTCTGCCACCGTTATAAGCCCGGGAGCTACTCTGGCAGTGATGAGGTAAACTCTCAGTG
D kyathit       AATTCCTCACAGCTCTGCCACCGTTATAAGCCTGGGAGCTACTCTGGCAGTGATGAGGTAAACTCCCAGTG
D tinwini       AATTCCTCACAGCTCTGCCACCGTTATAAGCCAGGGAGCTACTCTGGCAGTGATGAGGTAAACTCCCAGTG
                             L  C  H  P  Y  K  P  G  S  Y  S  G  S  D  E
```

```
pcexon3          T  P  R  P  P  A  V  T  T  T  G  R  L  D  G  R  .  .
BMGRA010000001  ACACCCAGGCCCCCAGCCGTCACCACCACCGGCCGCTTAGATGGCAGATAGTGAAGATGA Tuebingen
CABZ01020235    ACACCCAGGCCCCCAGCCGTCACCACCACCGGCCGCTTAGATGGCAGATAGTGAAGATGA Tuebingen
JAHVXS010000001 ACACCCAGGCCCCCAGCCGTCACCACCACCGGCCGCTTAGATGGCAGATAGTGAAGATGA AB
QQSU01000001    ACACCCAGACCCCCAGCCGTCACCATCACCGGCCGCTTAGATGGCAGATAGTGAAGATGA AB
JALCZT010090672 ACACCCAGACCCCCAGCCGTCACCACCACCGGCCGCTTAGATGGCAGATAGTGAAGATGA AB3
JALCZT010090680 ACACCCAGGCCCCCAGCCGTCACCACCACCGGCCGCTTAGATGGCAGGTAGTGAAGATGA AB3
JALCZS010003720 ACACCCAGACCCCCAGCCGTCACCATCACCGGCCGCTTAGATGGCAGATAGTGAAGATGA CG2
LKPD02012851    ACACCCAGACCCCCAGCCGTCACCATCACCGGCCGCTTAGATGGCAGATAGTGAAGATGA CG2
CALUEL010000207 ACACCCAGACCCCCAGCCGTCACCATCACCGGCCGCTTAGATGGCAGATAGTGAAGATGA SAT
JAIHOL010000484 ACACCCAGACCCCCAGCCGTCACCATCACCGGCCGCTTAGATGGCAGATAGTGAAGATGA SAT
JAIHOL010000647 ACACCCAGACCCCCAGCCGTCACCATCACCGGCCGCTTAGATGGCAGATAGTGAAGATGA SAT
BTXV01000001    ACACCCAGGCCCCCAGCCGTCACCACCACCGGCCGCTTAGATGGCAGGTAGTGAAGATGA NIES-R
D aesculapii    ACACCCAGACCCCCAGCCGTCACCACCACCGGCCGCTTAGATGGCAGATAGTGGAGATGA
D albolineatus  ACACCCAGACCCCCAGCCGTCACCACCACCGGCCGCTTAGATGGCAGATAGTGGAGATGA
D choprai       ACACCCAACCCCCCAGCCGTCACCACCACTGGCCGCTTAGATGGCAGATAGTGGAGATGA
D jaintianensis ACACCTAGACCCCCAGCCGTCACTACCACCGGCCGCTTAGATGGCAGATAGTGGAGATGA
D kyathit       ACACCCAGACCCCCAGCTGTCACCACCACCGGCCGCTTAGACGGCAGATAGTGGAGATGA
D tinwini       --ACCCAGACCCCCAGCCGTCACCACCACTGGCCGCTTAGATGGCAGATAGTGGAGATGA
```

Fig. S3. Zebrafish *scpplpq4*. Zebrafish scpplpq4 was identified based on similarities to the tiger barb *scpplpq4* in the encoded amino acid sequence. A premature termination codon (TGA) is found in pcexon 2 of *scpplpq4* in the Tuebingen and AB (GenBank, JAHVXS010000001, but not QQSU01000001) strains of *Danio rerio* but not in other strains of *Danio rerio* (shown at the end of the nucleotide sequence) or other *Danio* (D) species. Splice sites are highlighted in sky blue. In zebrafish, *scpplpq4* does not encode an Arg-Gly-Asp (RGD) sequence near the C-terminal. The region originally encoded RGD is highlighted in yellow.

```
scpp3: Genbank, JBEEIH010000015.1 (5562141..5562817) and CAAKHD010012036.1 (110067..110743).

                    pcexon1            pcexon2
F. commersonii      MKTVLVLGCLLCLALAHP MGIAHKRQARSNSGSDSNSDSRE
F. tabacaria        MKIVLVLVCLLCLALAHP MDIAHKRQARSNSGSDSNSDSSE
                    ** **** ********** *.******************* *

                    pcexon3
F. commersonii      VASMPDQLQFLQQLLKLIQQMNPPTTTPAATTTTAATTTTSAATTPTTTTTTTTQ
F. tabacaria        VAFMPDQLQFLQQLLKLIQQMNPPVTTSAATTTAAATTTTSAATTPTTTTTTTTQ
                    *** ********************.**.*****:*********************


scpp5: Genbank, JBEEIH010000015.1 (5566230..5567488) and CAAKHD010012036.1 (114688..115955).

                    pcexon1            pcexon2               pcexon3
F. commersonii      MKLALLCVCLVSSASASP SFLHYMPHFTGFRQVSPNQVS VQKAYIPGQSLPQTGVYSVEM
F. tabacaria        MKQALLCVYLVSTVYTSP SFLHYVPHFTGSRQVSPNQVS VQNAYIPGQSLPQTGAYSVEI
                    ** ***** ***:. :** *****:***** ********* **:************.****:

                    pcexon4          pcexon5                 pcexon6
F. commersonii      IYPNKFAGSVAGVKPVQ PLPSHGFIKYSIPQPTGRQSVEV YFPYDFSQQR
F. tabacaria        IHPNKFASGGAGVKPVQ PFASHGFIKYSIPQPAGRQSVEV YFPYDFSQQR
                    *:*****.. ******* *:.************:******* **********

                    pcexon7          pcexon8                      pcexon9
F. commersonii      VLPFEFPPQMITQQISN NSPFDANPPLSQNPTQSVQQDRTPQINQ MPATV
F. tabacaria        VLPFEFLPQIIPQQISN NPPFDANPPPSQNPSQSVQQEQPPQMNQ MPANV
                    ****** **:*.***** *.******* ****:*****::.**:** ***.*
```

Fig. S4. Amino acid sequences encoded by *scpp5* and *scpp3* in cornetfish. Both *scpp3* and *scpp5* were identified in two cornetfish species, *Fistularia commersonii* and *Fistularia tabacaria*. In the *F. commersonii* genome, *scpp3* and *scpp5* are located on chromosome 15 (Fig. 3) and comprise three pcexons and nine pcexons, respectively. SXE sequences (X represents any amino acids; Glu may be replaced by phospho-Ser), in which the first Ser residue is potentially phosphorylated, are shown in blue. *Fistularia commersonii* genes on chromosome 23: *spp1*, JBEEIH010000023.1 (12860960..12864290); *scpp8*, chromosome 23, JBEEIH010000023.1 (12868120..12869442, complement). *Fistularia tabacaria* genes: *spp1*, CAAKHD010012027.1 (36246..39111); *scpp8*, CAAKHD010012027.1 (44436..45205, complement). See Healey et al. (2024) for other SCPP genes.

```
enam: see Table S1 for genomic coordinates.

                pcexon1            pcexon2  pcexon3
surface         MKAVAIFLCFVAYTFAAP APDSGSQE QQIAAHANTALHLMELYRMYGHLQQQ
cave            MKAVAIFLCFVAYTFAAP APDSGSQE QQIAAHANTALHLMELYRMYGHLQQQ
                ****************** ******** **************************

                pcexon4     pcexon5    pcexon6
surface         GVAAPAPAPVQ AQAQSVQPQQ PFLFNYPTLPRGGDNSDEEAQ
cave            GVAAPAPAPVQ AQAQSVQPQQ PFLFNYPTLPRGGDNSDEEAQ
                *********** ********** *********************

                pcexon7
surface         PNYAAFYPPHGFPAVPAAPAAPAAPLNSDEAEGAEEAEAVEAEPAAEAEPAAEAEPAAEA
cave            PNYAAFYPPHGFPAVPAAPAAPAAPLNSDEAEGAEEAEAVEAEPAAEAEPAAEAEPAAEA
                ************************************************************


Surface         VPAAEAVPAVDPAVDLAVDPLVPVAAPIDPAVIDPHVAVEVGIEVPASANIPAADVLAAA
Cave            VPAAEAVPAVDPAVDLAVDPLVPVAAPIDPAVIDPHVAVEVGIEVPASANIPAADVLAAA
                ************************************************************


Surface         TAIAADPALVPQGAPVAIEIDTTVVGPEVGAPIVADSPVQPF
Cave            TAIAADPALVPQGAPVAIEIDTTVVGPEVGAPIVADSPVQPF
                ******************************************

scpp1: see Table S1 for genomic coordinates.

                pcexon1            pcexon2          pcexon3
surface         MKLTVVILCLLGATAANP ILHKVSMEIIDHASNS TSSVSESSEESNTSEHD
cave            MKLTVVILCLLGATAANP ILHKVSMEIIDHASNS TSSVSESSEESNTSEHD
                ****************** **************** *****************

                pcexon4    pcexon5      pcexon6        pcexon7
surface         SSPENTSENI SSESDESKSDEQ TSDLSHSHSLEER FGTGEPGMTTDNSQGSQENMRK
cave            SSPENTSENI SSESDESKSDEQ TSDLSHSHSLEER FGTGEPGMTTDNSQGSQENMRK
                ********** ************ ************* **********************

                pcexon8
surface         NWVHLINVKMASKEDTEEVTDQPDEEDKDTTEDQTSESSESTEKPTP-SSSSSSTEDSRA
cave            NWVHLINVKMASKEDTEEVTDQPDEEDKDTTEDQTSESSESTEKPTPSSSSSSSTEDSRA
                *********************************************** ************


surface         VVVDSSENSHSNSSSSSSSSESHESSHSTESTESQSKECPPGTDSNECDSDEYQFHDVGD
cave            VVVDSSENSHSN--SSSSSSESHESSHSTESTESQSKECPPGTDSNECDSDEYQFHDVGD
                ************  **********************************************


surface         DGATDPFNGFHTPDNAGHEFAFKR
cave            DGATDPFNGFHTPDNAGHEFAFKR
                ************************
```

Fig. S5. Amino acid sequences encoded by *enam* and *scpp1* in surface dwelling and Tinaja cave dwelling Mexican tetra. In Mexican tetra, *enam* and *scpp1* were identified in both a surface-dwelling population and a cave-dwelling population. Two Cys residues are shown in red, and Ser residues in Ser-Xaa-Glu (SXE, Xaa and X represent any amino acids; a phosphorylated Ser residue may replace the Glu residue) sequences, in which the Ser residue is potentially phosphorylated, are shown in blue. A dash represents an unaligned gap.

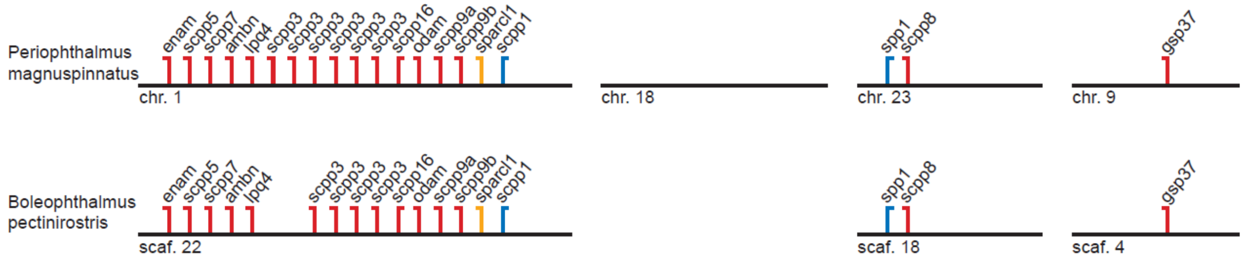

Fig. S6. SCPP genes in mudskippers. In the present study, 19 SCPP genes, including six *scpp3* genes, were identified on three different chromosomes (chr. 1, chr. 18, and chr. 9) in *Periophthalmus magnuspinnatus* (mudskipper) and 17 SCPP genes, including four *scpp3* genes, were found in three different scaffolds (scaf. 22, scaf. 18, and scaf. 9) in *Boleophthalmus pectinirostris* (great blue spotted mudskipper), as shown above (the location of SCPP gene cluster Ib was not uniquely determined and is now shown here). These results differ from the previous study that found nine SCPP genes and *sparcl1* in the genome sequence of three mudskippers (*Boleophthalmus pectinirostris*, *Periophthalmus magnuspinnatus*, and *Periophthalmus* modestus) (Bian *et al*., 2024; see the text). Although Bian *et al*. did not find *scpp7*, *ambn*, *scpp3*, or *gsp37*, all these genes were identified in both *Periophthalmus magnuspinnatus* and *Boleophthalmus pectinirostris* (Table S1). Furthermore, Bian *et al*. identified *scpp8*, *scpp11a*, and *scpp11b* (*scpp11b* was confused with *scpp3a*) on chromosome 22 in *Periophthalmus magnuspinnatus* and in scaffold 10 (not chromosome 10) in *Boleophthalmus pectinirostris*. However, I found three genes, *nrdc* (nardilysin), *larp1b* (la-related protein 1b), and *ltbp2* (latent tgf-beta-binding protein 2) in the region where *scpp8*, *scpp11a*, and *scpp11b* are reportedly present. None of these three genes are related to SCPP genes. Bien *et al*. also reported that, in *Boleophthalmus pectinirostris*, *enam* was found in scaffold 17 (not chromosome 17), while *scpp5* and other SCPP genes in cluster Ia (Fig. 2) were located in scaffold 22. In the genomic region where *enam* is reportedly located, I found a gene encoding a hypothetical protein, KAJ0058035.1. The hypothetical gene that encodes KAJ0058035.1 consists of a single protein-coding exon and differs from *enam*. In conclusion, mudskippers have various SCPP genes, and the reduction of scales in mudskippers is not associated with the repertoire of SCPP genes.

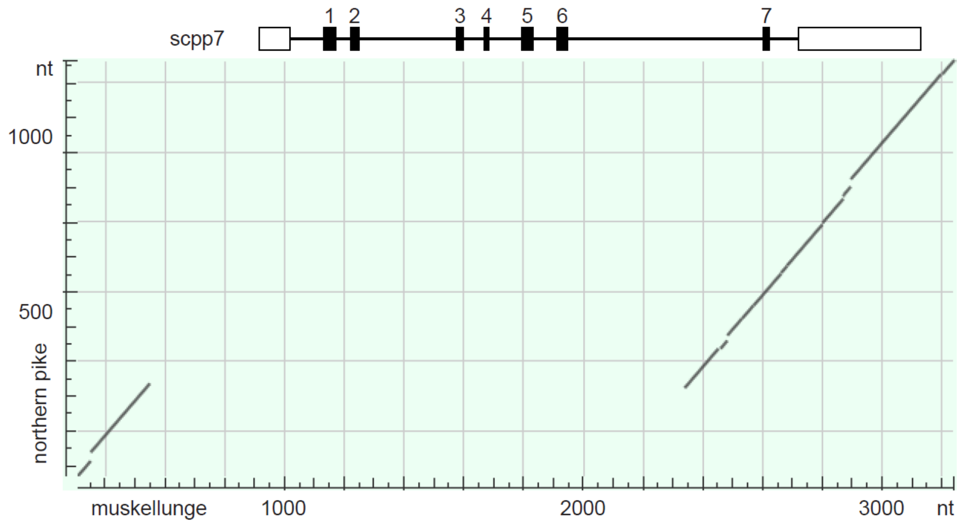


```
>scpp7_muskellunge
METSFLIASFLGTALCVPQMMFVDFDFHAANAQAHQAGQATPDLEIVLPVGGRVASGDFIKHEIPQANGQESVEI
YHPFRIQVAPAAPAAPAAPALTSDEEDE.
```

Fig. S7. Pike *scpp7* genes. Muskellunge *scpp7*, consisting of 7 protein-coding exons (pcexons; filled boxes) and 2 entirely non-protein-coding exons (open boxes), is shown on the top of the dot plot. The encoded amino acid sequence is shown below. The first amino acid encoded by 7 pcexons are highlighted in yellow. The diagonal lines in the dot plot show sequence similarities between a muskellunge genomic region containing *scpp7* and a corresponding genomic region of northern pike. The result shows a deletion of pcexon 1 – pcexon 6 of *scpp7* in the northern pike genome. In northern pike, only pcexon 7 and the entirely non-protein coding last exon remain in the genome. The dot plot was made using BLASTN at NCBI as described in the methods. Muskellunge *scpp7* was found in JACXGH010000720.1 (43778..47091).

```
(a)                      pcexon1                   pcexon2
Triplophysa dalaica      MDCLR-LALFGFILVTFTVCKP VRVHHTSSSSESSSSESSQSSSSEETQTLTLTESQSK
Triplophysa rosa         MDCWRLFALFGFILVTFTVCKP ITVHHTSSSSESSSSESSQSSSSEETRTPTPTESQSI

                         pcexon2
Triplophysa dalaica      NDQTPTIRRTIT.QTLEDMKIC-LSPDVSAQPDALDSYIRTSDPVSRGDNI
Triplophysa rosa         NENLQRANAQTPSTITGQKLEDMKLHHMSPPPDASDSNIRTSNPARRGDNI
```

(b)

```
>JAFHDT010000002.1:c34574127-34572975 Triplophysa rosa voucher Wulong-Trosa-837819 linkage group
LG2, whole genome shotgun sequence
GCCAAACATTTGATCTTTAGTGCTTGAAGTAGGCCATTTTCACATCTTTTCTGAGGAGTTTAAACACACGGTAAGTCTCACCATATCTGATGAAAAA
CACAGTTGTTGCTGTTATCTCGTTTAATAATGATTTTAATGAATGTAATGTCATTTTACCCTCTTTAACAGGGAAGGTCATTTAATAGAAGATGGAC
TGTTGGAGACTATTTGCACTATTTGGATTTATTTTAGTGACTTTCACCGTTTGTAAACCAGTACGAGAAAATGAAGCACTATTTTTTATTCAACAAA
TTAATATATTTCAATGTATATGATAGCTCTATTTCTTGTCACTGTAAAATAATGTTTTTTAATTAATTTTAATTGACATTTTATGCATTTAGCAAGT
GACTTCATCTATTTTTTGAATTCATCTATTTTTTCAGTTTTCCTTAGAGACCCATGACTTTGCATTTCTAAATTGCTGAATGTTTGTTTAAACATAA
CTAGACTTTTTATGCTTTATATCTTTGTAAGTCACTGGAGAGGATGCAGTTGCACCAGTGTATGAAATGTTCTGTTTGAATAAACCTTGACCGTTTT
AAATGGTCTTTTAACCGATTACTGTGCATCACACATCATCCAGTTCTGAATCTTCCAGTTCTGAATCCAGTCAGTCAAGCTCTTCAGAAGAGACTCG
AACTCCAACACCCACTGAATCACAGAGCATCAATGAGAATCTACAGCGGGCCAATGCTCAAACTCCAAGTACCATCACAGGACAAAAGCTGGAGGAC
ATGAAGCTCCACCACATGTCTCCTCCACCGGATGCTTCAGATTCAAACATCAGGACCAGTAACCCTGCCCGTCGAGGAGATAACATTTAATAACCCG
ACTAACCACAAAATGTATTCTGAGCTTGTTTTTTGTTAAAAATACAAATGCTTTATGCCATTTGTAAAGTTAATTCCAGTTAAAAGCATGTGATAAA
ATTCACTATATTTCTATAAACAACAAACATGTATGCAACTCATCCATTTGAGCAGTGAGATTGTTGAAGATGCACTGGCACAAGTGATTTTAGACAC
AAATTTCATGTTAAAACGATGTTTACTTATAATGGCTATACATAAAAACTAACTTTTATCCCAATTCAATAACTCCCATTTTGTTT
```

Fig. S8. ***Triplophysa gsp37*** **genes**. (**a**) Amino acid sequences encoded by *gsp37* in *Triplophysa dalaica* and *Triplophysa rosa*. The premature termination codon of *Triplophysa dalaica* is shown by a red "." and highlighted in yellow. A Cys residue in the mature protein and RGD integrin-binding sequences are shown in red. Clusters of potentially phosphorylated Ser (S) residues are shown in blue. (**b**) The genome sequence containing *Triplophysa* rosa *gsp37*. Exon sequences are shown in blue, and initiation and termination codons are shown in red. The splice donor and acceptor sequences are highlighted in yellow. Note the CG splice acceptor of pcexon 2. Based on the premature termination codon in *Triplophysa dalaica gsp37* and the nucleotide substitution at the splice acceptor of *Triplophysa rosa gsp37*, *gsp37* was considered to be non-functional in both *Triplophysa* species. The premature termination codon in *Triplophysa dalaica* was confirmed by SRX8097848, and the "CG" splice acceptor sequence was confirmed by SRX9863037. In addition to defects in *gsp37*, *scpp7* was identified in neither *Triplophysa dalaica* nor *Triplophysa rosa*.

(a)

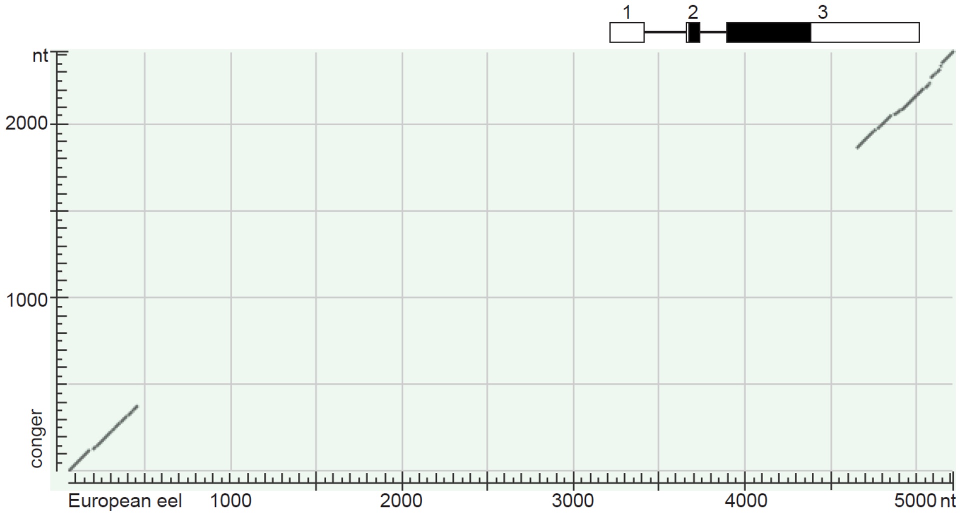

(b)

The last pcexon was found on chromosome 6.

```
>scpp7, last pcexon
GTGGGTCTACCCAAAGGAGACGATGACGACAGCCAGGATGACTAAAG
 V  G  L  P  K  G  D  D  D  D  S  Q  D  D  .
```

Fig. S9. Conger *gsp37* (a) and *scpp7* (b). (**a**) European eel *gsp37* (GenBank gene ID, LOC118206793) consists of 3 exons, shown by three boxes above the dot plot (white regions represent 5'- and 3'-untranslated regions, and black regions represent protein-coding regions). The diagonal lines in the dot plot show sequence similarities between a European eel genomic region containing *gsp37* and a corresponding conger genomic region, JAFJMO010000011.1 (3,195,927..3,198,060). The result shows a deletion of exon 1, exon 2, and a 5' half of exon 3, including the entire protein-coding region, of *gsp37* in the conger genome. The dot plot was made using BLASTN at NCBI as described in the methods. Although non-protein coding regions of the European eel genome sequence show nucleotide sequence similarities to the conger genome sequence (as shown by diagonal lines in the dot plot), the protein-coding sequence of *gsp37* did not detect nucleotide sequence similarities in the conger genome. This result suggests deletion of the *gsp37* protein-coding sequence. (**b**) Only the last pcexon of *scpp7* was identified in conger on chromosome 6, NC_083765.1 (28962755..28962799).

Supplementary Notes
Genes Encoding P/Q-rich SCPPs

SCPPLPQ8: This gene was found only in five otocephalans among teleosts: denticle herring, allis shad, zebrafish, Wuchang bream, and Mexican tetra. Like *scpplpq8* in non-teleost actinopterygians, Mexican tetra *scpplpq8* encodes a cluster of pSer residues by protein-coding exon (pcexon) 2, an aromatic residue-rich sequence by pcexon 3, and a Thr- and Pro-rich low complexity sequence and an Arg-Gly-Asp (RGD) integrin-binding sequence near the C-terminal by the last pcexon. An RGD sequence is encoded by all five teleost *scpplpq8*, while some other sequence elements are missing in some teleost *scpplpq8* genes. The C-terminal amino acid sequences, including RGD, encoded by bichir, gar, bowfin, zebrafish, and Wuchang bream scpplpq8 genes show similarities.

ENAM (*fa93e10*, *si:dkey-22i16.3*, *wu:fa93e10*): *ENAM* encodes one of the three principal enamel matrix proteins in mammals. Similarly, *enam* encodes an enamel matrix protein in gar and is thought to be the most ancient enamel matrix protein in vertebrates (Kawasaki et al., 2023). In most teleosts, *enam* encodes one or more potentially phosphorylated Ser residues (the Ser residue in Ser-Xaa-Glu (SXE) sequences; referred to below as a pSer residue) by pcexon 2, the penultimate pcexon, and the last pcexon. The region C-terminal to the last pSer residue is acidic, rich in Glu residues. In Mexican tetra, an *enam-like* (*enaml*) gene is found in cluster Ib. The *enaml* gene also encodes these pSer residues and the following acidic region. This acidic structure resembles 32-kDa enamelin, which is encoded by mammalian orthologs and known to show a high affinity for hydroxyapatite (Tanabe et al., 1990). One or more premature termination codons are found in zig-zag eel and Asian swamp eel *enam* genes. However, these genes are not considered non-functional in this study because many RNA-seq reads are found.

SCPP5 (*si:dkey-22i16.4*): This gene is found only in actinopterygians and encodes an enamel matrix protein in gar (Kawasaki et al., 2021). In all teleosts, *scpp5* encodes two or three pSer residues in Ser-Val-Glu-Leu sequences at the 3’ end of exons (Val and Leu may be replaced by hydrophobic amino acids, Val, Leu, Ilu, Met, Tyr, or Phe). The only exception is Siluriformes *scpp5* genes, which encode one of the three pSer residues in a Ser-Ilu-Glu sequence at the 3’ end of pcexon 5. Similar exons are also found in bichir, gar, and bowfin *scpp5* genes.

SCPP7 (*si:dkey-22i16.5*): This gene is found only in actinopterygians. Like *scpp5*, exons encoding pSer near the 3’ end are found in various teleosts, although this exon is not found in most Percomorpha species. Similar exons are also found in bichir, gar, and bowfin *scpp7* genes. In teleosts, *scpp7* also encodes a Pro- and Ala-rich or Pro-, Ala-, and Val-rich low complexity sequence by the penultimate pcexon, and a cluster of acidic residues by the last pcexon. In tarpon, Mexican tetra, and red-bellied piranha, an scpp7-like gene (*scpp7l*), which reside in cluster Ib, is found. All these genes have two exons encoding pSer near the 3’ end. Although the last pcexon encodes acidic residues, basic residues are equally encoded by Mexican tetra and piranha *scpp7l* genes, and the exon corresponding to the penultimate exon of *scpp7* is missing in Mexican tetra and piranha *scpp7l* genes.

AMBN (*scpp6*, *si:dkey-22i16.6*): *AMBN* also encodes one of the three principal enamel matrix proteins in humans. In gar, the secretion of the Ambn protein begins late during the secretory stage of enamel, and Ambn distributes on the surface layer of enamel (Kawasaki et al., 2023). In tarpon, two pSer clusters are encoded by the 3’ end of pcexon 2 and by a small penultimate exon, which is common to non-teleost actinopterygians and sarcopterygian *AMBN* genes. However, such pcexon 2 was not identified in all the other teleost *ambn* genes investigated. By contrast, the last six amino acids encoded by the penultimate pcexon of *ambn* is pSer-pSer-Glu-Glu-Xaa-Yaa (Xaa and Yaa represent any amino acids) in all investigated

teleost. This characteristic penultimate exon is found in *AMBN* of non-teleost actinopterygians and sarcopterygians, including humans.

SCPPLPQ4: This gene is found only in actinopterygians. Originally, *scpplpq4* comprised three pcexons. Among these pcexons, pcexon 2 encodes a cluster of pSer, and pcexon 3 encodes a low complexity sequence rich in Pro and Ala or Thr in the middle portion and RGD near the C-terminal. Similar pcexon 2 and pcexon 3 are also found in *scpp3*. The difference between *scpplpq4* and *scpp3* is the presence or absence of RGD in the encoded amino acids with the exception of *scpplpq4* in stone loach and zebrafish, and by one of the three copies of *scpplpq4* in cardinalfish. Although these three genes do not encode RGD, these genes were identified as *scpplpq4* by similarities in encoded amino acid sequences to *scpplpq4* in phylogenetically close species or in the same species. Among zebrafish, a premature termination codon is present in pcexon 2 of *scpplpq4* in the Tubingen strain and one of the two genome assemblies of the AB strain (Fig. S3).

SCPP3: This gene is found in all actinopterygians except syngnathids. Furthermore, three or more *scpp3* genes are clustered in the genome of most actinopterygians. The most common *scpp3* genes comprise two small pcexons and a relatively small last pcexon. Small sizes of this gene may contribute to frequent gene duplication events. In addition, many duplicate *scpp3* genes often show significant expression levels (Table S1), which likely contribute to high levels of protein production as described in the main text. Multiple *scpp3* copies may have been selected to meet the requirement of high protein levels. In most *scpp3* genes, a cluster of pSer residues is encoded by pcexon 2, while a low complexity sequence is encoded by the last pcexon. The number of amino acids encoded by the last pcexon of *scpp3* genes varies from 25 (tuna) to 492 residues (pike). Some *scpp3* genes are highly derived from the common *scpp3* genes. In European eel, pcexon 2 was duplicated, and the duplicated pcexon 2 expanded by intraexonic duplication and encodes 285 residues, most of which constitute pSer-Ala-Glu repeats. Although this and other highly derived *scpp3* genes are found in various teleosts, these derived genes are also referred to as *scpp3* in the present study.

SCPP16: This gene is found only in teleosts and *Polypterus*, and *Erpetoichthys* among non-teleost actinopterygians. Coelacanth *scpppq2* (Kawasaki and Amemiya, 2014) may be orthologous to *scpp16*. In the SCPP gene cluster Ia, most SCPP genes are arranged in the same direction except *scpp16*. This gene comprises many small pcexons, and frequent duplications and deletions of small exons make it highly difficult to identify all pcexons composing *scpp16* unless RNA-seq data are available.

ODAM (*scpp2*, *scpp4*): This gene was first identified in fugu and named *scpp2*. In zebrafish, the expression of *odam* begins late after the cap enameloid matrix is formed (Kawasaki, 2009). Similarly, *ODAM* is expressed during the maturation stage of enamel formation in mammals (Moffatt, 2007). In most teleosts, pcexon 2 of *odam* encodes Ala-pSer-Asn-pSer-Asn-Glu at the 3’ end. Similarly, in various non-teleost actinopterygians and sarcopterygians, pcexon 2 of *ODAM* encodes pSer-Ala-pSer-Asn-pSer-Asn-Glu at the 3’ end. Except for elopomorphs (tarpon, European eel, and conger), the penultimate pcexon of *odam* encodes RGD. The penultimate exon of *ODAM* encoding RGD is found in bowfin but has not been found in other bony vertebrates.

SCPP9A (*scpp9*): This gene is found in teleosts, bowfin, and gars. After *scpp9* (*scpp9a*) was identified in zebrafish, an *scpp9*-like (*scpp9b*) gene was found in gar. In zebrafish, *scpp9b* was missing, while *scpp9a* was located in unassembled gap and not identified in spotted gar. For this reason, gar *scpp9b* was initially thought to be orthologous to *scpp9a*. The penultimate exon of *scpp9a* encodes Arg at the first position, which is found in all investigated teleosts, bowfin, and gars. A small pcexon 2 of *scpp9a* encodes four to

six amino acids (mostly five amino acids) in all investigated teleosts. The last pcexon of *scpp9a* encodes a Cys residue in most euteleosts, but not in other teleosts. A Cys residue is encoded by the penultimate pcexon in elopomorphs. In tarpon and European eel, an *scpp9a*-like (*scpp9al*) gene is found in the SCPP gene cluster that derived from cluster Ib. A small pcexon 2 encoding five amino acids and the penultimate pcexon encoding a Cys residue are also found in *scpp9al*.

SCPP9B: This gene is found only in actinopterygians, including all non-teleost actinopterygians studied to date, elopomorphs, an osteoglossomorph, and most euteleosts, except syngnathids and ocean sunfish, but only in Clupeiformes among otocephalans. Surprisingly, *scpp9b* lost all introns and became a single exon gene in the common ancestor of Acanthomorpha in the Euteleostei lineage. In non-teleost actinopterygians, pcexon 2 of *scpp9b* encodes a pSer-Xaa-pSer-Yaa-Glu or pSer-Xaa-Glu sequence at the 3’ end. A similar pcexon 2 is found in most teleosts, except delta smelt, Asian icefish, and Acanthomorpha (because pcexon 2 is not present in Acanthomorpha). However, two pSer residues are encoded at a similar or the same positions in all teleost *scpp9b* genes studied.

SCPP8: This gene is considered one of the two co-orthologs of *scpplpq14* that is found in bowfin, gars, sturgeon, and paddlefish. Like *scpplpq14*, *scpp8* comprises only two pcexons, and the last pcexon encodes one or more pSer clusters, and RGD near the C-terminal. Although a Cys residue is also encoded in the last pcexon slightly upstream of RGD in all non-teleost ray-finned fish, the Cys residue is not encoded by the last pcexon of *scpp8* in some teleosts. In European eel, conger, and some otocephalans, *scpp8* is duplicated or triplicated. In these duplicated or triplicated *scpp8* genes, the pSer cluster or the RGD sequence may be substituted.

GSP37: Like *scpp8*, *gsp37* is considered one of the two co-orthologs of *scpplpq14*. Like *scpplpq14*, *gsp37* comprises two pcexons, and the last pcexon encodes one or more pSer clusters near the C-terminal, a Cys residue usually near the C-terminal, and RGD close to the C-terminal end except for *gsp37* of pond loach (no Cys) and spotted parrotfish (no RGD). The Cys residue is encoded near the N-terminal pSer cluster in some teleosts.

SCPPLPQ8: This gene is found in non-teleost actinopterygians and only five otocephalans among teleosts. All *scpplpq8* genes comprise four to six small pcexons and a relatively large last pcexon that encodes RGD near the C-terminal. In non-teleost actinopterygians, pcexon 2 of *scpplpq8* encodes a pSer-Xaa-pSer-Yaa-Glu sequence, but pcexon 2 encoding pSer cluster is found only in Mexican tetra among teleosts. Amino acid sequence similarities are found in a Tyr-rich amino acid sequences encoded by pcexon 3 and pcexon 4 of zebrafish *scpplpq8* and encoded by pcexon 5 and pcexon 6 of spotted gar *scpplpq8*. C-terminal amino acid sequences, including RGD, encoded by zebrafish pcexon 5 and spotted gar *scpplpq8*, are also similar to each other.

SCPPLPQ5: This gene is found in most non-teleost actinopterygians (except bowfins), elopomorphs, an osteoglossomorph, and all otocephalans investigated, but only in Salmoniformes among euteleosts. In bichirs and gars, *scpplpq5* encodes a cluster of pSer residues by pcexon 2 and the penultimate pcexon and an RGD sequence by the last pcexon near the C-terminal. While the penultimate pcexon encoding a pSer cluster is found in all teleosts *scpplpq5* genes, pcexon 2 encoding a pSer cluster is not found in teleosts. Instead, a pSer-Xaa-Glu sequence is encoded by the 5’end of the last pcexon in various species. The RGD sequence near the C-terminal is encoded by only tarpon, elephantfish, denticle herring, and allis shard *scpplpq5* genes. Similarities in amino acid sequences encoded by gar and tarpon *scpplpq5* genes are easily detectable.

SCPP17: This gene has been found upstream of *scpplpq5* in only two otocephalans, denticle herring and Mexican tetra. The ortholog of *scpp17* has not been identified in non-teleost actinopterygians. In both denticle herring and Mexican tetra, *scpp17* comprises small pcexon 2 encoding a cluster of pSer residues, and large pcexon 3, which is followed by two or more small pcexons. Among gar and bichir SCPP genes located downstream of *scpplpq5*, only *odam* and *scpplpq3* (showing similarities to *odam*) possess similar small and large pcexons. The amino acid sequence encoded by pcexon 2 and 5'-end of pcexon 3 by Mexican tetra *scpp17* and that encoded by pcexons 2 and 5'end of pcexon 3 of bowfin *scpplpq3* show low similarities, suggesting potentially orthologous relationship of *scpp17* in teleosts and *scpplpq3* in non-teleost actinopterygians.

SCPP11A and SCPP11B: These two genes are found only in five otocephalans and two euteleosts and are arranged in a head-to-head orientation (always found as a pair of genes). The ortholog of *scpp11a* and *scpp11b* has not been confirmed in non-teleost actinopterygians. Both *scpp11a* and *scpp11b* comprise two small pcexons and a relatively large last pcexon. Although pcexon 2 of soldierfish *scpp11a* encode pSer-Xaa-Glu sequence at the 3' end, all the other *scpp11a* and *scpp11b* genes do not encode any pSer residues. The last pcexon of *scpp11a* and *scpp11b* encodes Tyr-rich sequence.

SCPP13 and SCPP14: These two genes are found also in a head-to-head orientation only in the five otocephalans and two euteleosts that possess both *scpp11a* and *scpp11b*. In addition, only *scpp13* is found in denticle herring. Both *scpp13* and *scpp14* comprise two small pcexons and one relatively large last pcexon like *scpp11a* and *scpp11b*. However, unlike *scpp11a* or *scpp11b*, *scpp13* and *scpp14* encode a pSer-Xaa-Glu sequence except for denticle herring *scpp13* and delta smelt and soldierfish *scpp14* genes. Amino acid sequences encoded by pcexon 2 of *scpp13* and *scpp14* show similarities, and similar amino acid sequences are also encoded by gar and bichir *scpplpq9* genes. The most parsimonious scenario that explains the relationship of these genes is that *scpp13* and *scpp14* arose from an *scpplpq9* ortholog by an inverted duplication in the teleost lineage.

SCPP12: This gene is found upstream of *gsp37* in the five otocephalans and two euteleosts that also possess *scpp11a*, *scpp11b*, *scpp13*, and *scpp14*. The *scpp12* gene comprises only two pcexons, and similarities of amino acid sequences among the teleost orthologs are limited to a region encoded by 5' end of *scpp12*. In addition, amino acid sequences encoded by allis shad and soldierfish *scpp12* genes are also similar near the C-terminal, and this region also shows similarities to gar *scpplpq12*, which also comprises two pcexons, like *scpp12*. In the genome of gars, *scpplpq12* is located upstream of *scpplpq14*, which is considered the ortholog of *gsp37* (and also *scpp8*). The arrangement of *gsp37* and *scpp12* is thus similar to the arrangement of *scpplpq14* and *scpplpq12* (Fig. S2). It is highly likely that teleost *scpp12* genes are orthologous to *scpplpq12* in non-teleost actinopterygians.

The *scpp12-gsp37* cluster is separated from the *scpp13-scpp14* cluster by three to six genes that are not related to SCPP genes in six out of seven teleost species. These four genes are thus considered a single SCPP gene cluster. However, the original cluster must have underwent local rearrangement because SCPP gene cluster II is uninterrupted by other genes in gars and bichirs.

SCPPLPQ20: This gene is found in all non-teleost actinopterygians but only in elopomorphs and an osteoglossomorph among teleosts. The expression of Senegal bichir *scpplpq20* was detected in the dental epithelium (Delgado et al., 2023). In all ray-finned vertebrate SCPP genes, *scpplpq20* is the only gene that does not form a gene cluster. All *scpplpq20* genes comprise many small pcexons (10-13 pcexons in teleost *scpplpq20* genes and 17-20 pcexons in non-teleost ray-finned vertebrate *scpplpq20* genes). Among these exons, four or more pcexons encode Ser-Ile-Glu-Ile at the 3' end (Ile may be replaced by Leu, Val, Met,

Tyr, or Phe in most cases). Similar exons are also found in *scpp5* and *scpp7* genes. In teleosts, the last pcexon of *scpplpq20* encodes a Cys residue.

Delgado S, Fernandez-Trujillo MA, Houee G, Silvent J, Liu X, Corre E, Sire JY. 2023. Expression of 20 SCPP genes during tooth and bone mineralization in Senegal bichir. *Dev Genes Evol* 233:91-106. https://doi.org.10.1007/s00427-023-00706-w

Genes Encoding Acidic SCPPs

SCPP1: This gene is found in all actinopterygians studied to date but not in sarcopterygians. In all actinopterygians, *scpp1* comprises several small pcexons and a large last pcexon, and many pSer-Xaa-Glu sequences are encoded by most of these pcexons. In most actinopterygians, the last pcexon encodes at least two Cys residues, separated by 5-13 amino acids. Furthermore, these Cys residues are followed by an Ilu/Val-Gly-Asp-Asp (I/VGDD) sequence, except elopomorphs and basal otocephalans (denticle herring and allis shad). An *scpp1*-like (*scpp1l*) gene is found in cluster Ib in elopomorphs, an osteoglossomorph, some otocephalans, and various euteleosts. However, two Cys residues and an I/VGDD sequence are encoded only by the last pcexon of tarpon *scppl1l*. Two Cys residues are encoded by the last pcexon of *scpp1l* in European eel, conger, elephantfish, and otocephalans, but only salmoniforms among euteleosts. In all investigated euteleosts, *scpp1l* encodes continuous Ser residues, which do not always form pSer-Xaa-Glu sequences.

IBSP: This gene is found in both ray-finned and sarcopterygians. In teleost, *ibsp* is found in cluster Ib in elopomorphs, an osteoglossomorph, and all investigated otocephalans, but only salmoniforms, soldierfish, and two wrasses among euteleosts. In most otocephalans and euteleosts, *ibsp* comprises two small pcexon and a large last exon. In non-teleost actinopterygians, by contrast, ibsp comprises two to four small pcexons, one middle-size penultimate pcexon, and the last large pcexon. This exon-intron structure suggests the loss of the middle-size penultimate pcexon in teleosts. The large last exon of *ibsp* encodes a Gly- and Glu-rich highly acidic low complexity sequence. The *ibsp*-like (*ibspl*) gene is found in cluster Ib in elopomorphs and an osteoglossomorph. In elopomorphs, *ibspl* comprises two small pcexons, a middle-size penultimate pcexon, and a large last pcexon. The middle-size penultimate exon was not found in elephantfish *ibsp*. In all these *ibsp* genes, the large last pcexon encodes RGD close to the 5' end, which is also found in bowfin *ibsp*, and a Glu- and Asp-rich highly acidic low complexity sequence.

MEPE: This gene is found in both ray-finned and sarcopterygians. Among teleosts, *mepe* is found only in tarpon and elephantfish. In most actinopterygians, except bowfin, *mepe* comprises four pcexons, and pcexon 2 encodes a Cys residue (not all pcexons of *mepe* have been identified in bowfin). Teleost, gar, and bichir *mepe* genes encode a relatively high density of charged amino acids in the N-terminal and C-terminal sequences. The amino acid sequences encoded by *mepe* are relatively well-conserved among actinopterygians.

DMP1: This gene is found in both ray-finned and sarcopterygians. In elopomorphs and otocephalans, *dmp1* comprises two or three small pcexons and a large last pcexon. The large last pcexon encodes a putative BMP1 cleavage site (MGQDD and its derivatives) in tarpon and some otocephalans. In euteleosts, the last intron is located between the second and third nucleotides of a codon (phase-2 intron), unlike most other introns in SCPP genes. It appears that the phase-2 intron was introduced within the last pcexon in the Euteleostei lineage. In teleosts, *dmp1* encodes many scattered pSer-Xaa-Glu sequences, and sequence similarities in the encoded amino acid sequence are generally low across phylogenetically distant species. However, tarpon, European eel, and allis shad *dmp1* genes show similarities to bowfin

dmp1 in the amino acid sequence encoded by pcexon 2, while allis shad *dmp1* shows similarities to other Otocephala *dmp1* genes in the amino acid sequence encoded by the last pcexon.

DSPPL1: This gene is found in various bony vertebrates but not in mammals. Among actinopterygians, *dsppl1* is found in all non-teleost actinopterygians (bowfin dsppl1 is found in latest version of genome assembly; NC_089861.1 (3923002..3926455)). In teleosts, *dsppl1* has been identified in elopomorphs, and an osteoglossomorph, otocephalans except cypriniforms, and salmoniforms among euteleosts. Teleost *dsppl1* genes comprise one, two, or three small pcexons and a large last exon. The 3' half of the large last exon encodes Ser-Ser-Asp repeats, poly-Ser, or their derivatives in all *dsppl1* genes found in teleosts. A BMP1 cleavage site is encoded by some *dsppl1*. If present, the BMP1-cleavage site is located upstream of the Ser-Ser-Asp repeats, as found in tetrapod orthologs.

SPP1: This gene is found widely in ray-finned and sarcopterygians except frogs. The penultimate pcexon and the last pcexon is large in size. The RGD sequence is encoded by the large penultimate exon in most teleosts, except Asian swamp eel (substituted to His-Gly-Asp), all non-teleost actinopterygians, and sarcopterygians studied to date.
